# A new candidate tumor suppressor tRF-Ser inhibits gastric cancer progression by regulating the CNBP/HSPA8 axis

**DOI:** 10.1038/s41419-026-08608-1

**Published:** 2026-03-25

**Authors:** Jian Jiao, Guangchuan Wang, Jin Liu, Kun Xiao, Zi Gao, Daocong Dong, Keshu Shan, Huaiping Cui, Liang Shang, Leping Li, Chunqing Zhang

**Affiliations:** 1 Department of Gastroenterology, Shandong Provincial Hospital Affiliated to Shandong First Medical University, Shandong First Medical University, Jinan, Shandong Province China; 2 Department of Gastrointestinal Surgery, Shandong Provincial Hospital Affiliated to Shandong First Medical University, Jinan, Shandong Province China

**Keywords:** Gastric cancer, Gastric cancer

## Abstract

Gastric cancer (GC) is a highly aggressive malignancy with a poor prognosis. Transfer RNA-derived small RNAs (tsRNAs) are implicated in tumorigenesis, but their precise mechanistic roles in GC progression remain incompletely understood. We performed high-throughput sequencing in four paired GC/normal tissues to profile tsRNAs. The functional and mechanistic role of a candidate tsRNA was systematically investigated, alongside a suite of techniques including fluorescence in situ hybridization, RNA immunoprecipitation, RNA pull-down, chromatin immunoprecipitation, and luciferase reporter assays. We identified a novel tsRNA, tRF-Ser, that was significantly downregulated in GC tissues and cell lines, and its expression was correlated with favorable survival. Functionally, tRF-Ser acted as a tumor suppressor by inhibiting epithelial-mesenchymal transition (EMT), inducing ferroptosis, and enhancing sensitivity to 5-fluorouracil chemotherapy. Mechanistically, tRF-Ser directly bound to the cellular nucleic acid-binding protein CNBP (a transcription factor), promoting its accumulation in the cytoplasm and preventing its binding to the HSPA8 promoter to downregulate HSPA8. Then, the tRF-Ser/CNBP/HSPA8 axis suppressed EMT by inhibiting β-catenin nuclear translocation and promoted ferroptosis by facilitating STUB1-mediated ubiquitination degradation of GPX4. Our study unveils that the tRF-Ser/CNBP/HSPA8 axis may constrain GC progression by regulating energy metabolism, which highlights the therapeutic potential of targeting this axis for GC treatment.

## Introduction

GC is a common gastrointestinal malignancy worldwide, characterized by high incidence and a grim prognosis [1, 2]. Chemotherapy, a cornerstone treatment for cancer [3], is often thwarted by the development of chemoresistance [4]. This resistance is driven by diverse mechanisms, including alterations in tumor-initiating stem-like cells [5], protective autophagy [6], and activation of drug efflux pumps [7].

tsRNAs, comprising tRNA-derived fragments (tRFs) and tRNA-derived stress-induced small RNAs (tiRNAs), are a novel class of non-coding RNAs (ncRNAs) generated by the specific cleavage of tRNAs [8]. These molecules are increasingly recognized as pivotal regulators of tumorigenesis. They can silence target mRNA via an miRNA-like mechanism [9] and participate in gene expression regulation through non-canonical pathways, such as interaction with RNA-binding protein [10] and interference with ribosome biogenesis [11]. Functionally, tsRNAs are crucial regulators of pathological processes ranging from tumor microenvironment remodeling to core malignant behaviors like proliferation, invasion, and metastasis. Their dysregulation is significantly correlated with advanced tumor stage, therapy resistance, and poor clinical outcomes [12–14]. Further, tRF-Val promotes GC progression by facilitating the nuclear translocation of EEF1A1 to suppress the p53 pathway [15], while tRF-29-79 inhibits lung cancer by modulating glutamine metabolism through the cytoplasmic translocation of PTBP1 [16]. These findings underscore the special roles of tsRNAs in oncology and their promising potential as therapeutic targets.

Intriguingly, the regulation of chemoresistance intersects with another critical process: ferroptosis [17]. This iron-dependent, non-apoptotic form of cell death, driven by lipid peroxidation, has become a central focus in cancer research [17, 18]. Tumor cells, with their frequent mutations, unique metabolic profile, and high reactive oxygen species (ROS) burden, are particularly susceptible to ferroptosis [19]. Further, key tumor suppressors like p53 [20] and BAP1 [21] are pivotal regulators of this process, directly linking ferroptosis to tumor progression and therapy response. Notably, the strategic induction of ferroptosis has shown promising preclinical efficacy in overcoming resistance in refractory tumors [17]. However, despite the established roles of tsRNAs in oncogenesis [15, 16] and ferroptosis [22], and the clear importance of ferroptosis in chemosensitivity response [17], a potential connection between tsRNAs and the regulation of ferroptosis and chemosensitivity remains a significant and unexplored frontier. Unraveling this link is critical for developing novel combination therapies.

In this study, we identified a specific tsRNA, tRF-1:16-Ser-CGA-4 (hereafter tRF-Ser), that was significantly downregulated in GC. We discovered that tRF-Ser inhibited EMT, activated ferroptosis, and sensitized GC cells to 5-fluorouracil (5-FU) chemotherapy. Mechanistically, we identified a novel pathway in which tRF-Ser bound to the transcription factor CNBP, modulating its nuclear localization and subsequent activation of the HSPA8 gene. Then, the tRF-Ser/CNBP/HSPA8 axis inhibited β-catenin-mediated EMT and promoted STUB1-dependent ubiquitination degradation of GPX4 to drive ferroptosis. Our findings suggest that the tRF-Ser/CNBP/HSPA8 axis may function as a key regulatory network in GC and a promising target for therapeutic intervention.

## Materials and Methods

### Clinical specimens and cell culture

We collected 90 paired GC and adjacent normal tissues, with all participants providing written informed consent. Surgical specimens were immediately snap-frozen in liquid nitrogen and stored at −80 °C. Three GC cell lines (HGC-27, AGS, and MKN-45) and two control cell lines (normal human gastric mucosal epithelial cells (GES-1) and human embryonic kidney cells (HEK-293T)) were sourced from the Culture Collection of the Chinese Academy of Sciences (Shanghai, China). All cells were authenticated by short tandem repeat profiling and tested free from mycoplasma. GC cell lines were cultured in RPMI-1640 medium (Cat No. C11875500BT, Gibco, USA), while control cells in DMEM (Cat No. C11995500BT, Gibco), both containing 10% fetal bovine serum (Cat No. A5256701, Gibco) and 1% penicillin-streptomycin (Cat No. P1400, Solarbio, China) at 37 °C with 5% CO₂.

### RNA sequencing

Small RNA sequencing (focusing on tRFs/tiRNAs) was performed for four paired GC and adjacent normal tissues by Aksomics (Shanghai, China), whereas transcriptome sequencing of tRF-Ser-overexpressing MKN-45 cells was carried out by Novogene (Beijing, China).

### Quantitative real-time polymerase chain reaction (qRT-PCR)

Total RNA extraction used RNA-easy isolation reagent (Cat No. R701-01, Vazyme, China), with cDNA synthesis following the manufacturer’s protocol (Cat No. AG11745, AG, China; Cat No. R323-01, Vazyme). qRT-PCR analyses were performed with SYBR Green master mix (Cat No. Q711-02, Vazyme), using β-actin and U6 as endogenous controls for mRNA and tRF-Ser, respectively. The 2⁻^ΔΔCt^ method was applied for quantification, with all primer sequences (Supplementary Table 1) synthesized by Sangon Biotech (Shanghai, China).

### Western blot (WB)

Protein extraction was performed with RIPA lysis buffer (Cat No. R0020, Solarbio) with phenylmethylsulfonyl fluoride (PMSF), followed by BCA quantification (Cat No. PC0020, Solarbio). Samples were resolved by SDS-PAGE and transferred to PVDF membranes. After blocking (5% skim milk), membranes were probed with primary antibodies (4°C, overnight) and corresponding secondary antibodies (1 h, room temperature). Signals were visualized using ECL substrate (Cat No. P10300, NCM Biotech, China). (Uncropped blots in Supplementary Fig. 8) The following antibodies were used: anti-E-cadherin (Cat No. 60335-1-Ig, RRID: AB_2881444, Proteintech, China), anti-N-cadherin (Cat No. 66219-1-Ig, RRID: AB_2881610, Proteintech), anti-β-catenin (Cat No. 66379-1-Ig, RRID: AB_2857358, Proteintech), anti-MMP-9 (Cat No. AB76003, RRID: AB_1310463, ABCAM, USA), anti-MMP-2 (Cat No. ab92536, RRID: AB_10561597, ABCAM), anti-Vimentin (Cat No. 60330-1-Ig, RRID: AB_2881439, Proteintech), anti-c-Myc (Cat No. ab32072, RRID: AB_731658, ABCAM), anti-Cyclin D1 (Cat No. 26939-1-AP, RRID: AB_2880691, Proteintech; Cat No. 2922, RRID: AB_2228523, CST, USA), anti-Snail (Cat No. sc-271977, RRID: AB_10709902, Santa, USA), anti-Twist (Cat No. ab50887, RRID: AB_883294, ABCAM), anti-HSPA8 (Cat No. 10654-1-AP, RRID: AB_2120153, Proteintech), anti-GPX4 (Cat No. ab125066, RRID: AB_10973901, ABCAM; Cat No. 67763-1-Ig, RRID: AB_2909469, Proteintech), anti-STUB1 (Cat No. 68407-1-Ig, RRID: AB_3085124, Proteintech; Cat No. ab134064, RRID: AB_2751008, ABCAM), anti-CNBP (Cat No. 67109-1-Ig, RRID: AB_2882413, Proteintech; Cat No. 14717-1-AP, RRID: AB_2081548, Proteintech), anti-RPS16 (Cat No. 15603-1-AP, RRID: AB_2180168, Proteintech), anti-CST6 (Cat No. 17076-1-AP, RRID: AB_2878345, Proteintech), anti-β-actin (Cat No. 20536-1-AP, RRID: AB_10700003, Proteintech; Cat No. 66009-1-Ig, RRID: AB_2687938, Proteintech), anti-Histone-H3 (Cat No. HY-P80166, RRID: AB_3102325, MCE, USA), anti-Ubiquitin (Cat No. sc-8017, RRID: AB_628423, Santa).

### Cell transfection

tRF-Ser mimics, inhibitors with matched negative controls, tRF-Ser-overexpressing lentivirus, and CNBP/HSPA8-targeting siRNAs were synthesized by General Biol (Anhui, China). HSPA8-overexpressing (pcDNA3.1) and three lentiviral plasmids (sh-tRF-Ser (pLent-U6), OE-CNBP (pLent-EF1a), and sh-CNBP (pLent-U6)) were constructed by Abiotech (Shandong, China).

For transfection: mimics/inhibitors and their controls, plasmids, and siRNAs were transfected using Lipofectamine 3000 (Cat No. L3000015, Thermo Fisher Scientific, USA), while lentiviral infections were performed with polybrene (Cat No. H8761, Solarbio) to enhance efficiency. Stable cell lines were established through puromycin (Cat No. HY-B1743, MCE) selection. All oligonucleotide sequences are provided in Supplementary Table 2.

### CCK-8 assay, colony formation assay, and wound healing assay

Cell viability was assessed by the CCK-8 assay: 3×10³ cells/well in 96-well plates were treated with a 10 μL CCK-8 (Cat No. CK04, DojinDo, Japan) at each time point (0, 24, 48, 72, 96 h), incubated for 2 h (37 °C, dark), and measured at 450 nm.

For the colony formation assay, 1 × 10³ cells/well in 6-well plates were cultured for 10–14 days. Fixed (4% paraformaldehyde, Cat No. BL539A, Biosharp, China) and stained (crystal violet, Cat No. G1014, Servicebio, China) colonies were counted manually.

The wound healing assay used confluent monolayers (>90%) in 6-well plates scratched with 10 μL tip. Wound closure was imaged at 0/48 h under a microscope (Olympus, Tokyo, Japan), with closure area quantified.

### Transwell migration and invasion assay

Cells were seeded in serum-free medium into Transwell chambers (8 μm pores; Cat No. 3422, Corning, USA), with Matrigel (Cat No. 0827045, ABW, China) coating for invasion assays or without coating for migration assays. The lower chamber contained complete medium, and after 48 h incubation at 37 °C, transmembraned cells were fixed (4% paraformaldehyde) and stained (crystal violet), then imaged and quantified under a microscope.

### Cell cycle detection

Cells were processed with a cell cycle detection kit (Cat No. CCS012, Multisciences Biotech, China) according to the manufacturer’s protocol. Briefly, after trypsinization and centrifugation, cell pellets were resuspended in 1 mL DNA staining solution with 10 μL permeabilization solution, incubated for 30 min at room temperature in the dark, and then analysis was conducted on a CytoFLEX S flow cytometer (Beckman Coulter).

### ROS assay

ROS level was assessed using an ROS detection kit (Cat No. C1300-2, Applygen, China). Briefly, cells were incubated with 10 μM probe in medium for 30 min at 37 °C/5% CO₂, then immediately imaged under a microscope. Signal intensity was quantified to reflect ROS level.

### Malondialdehyde (MDA) assay and Glutathione (GSH) assay

MDA and GSH levels were measured in lysates using the following kits: an MDA detection kit (Cat No. BC0025, Solarbio) and a GSH assay kit (Cat No. A006-2-1, Nanjing Jiancheng Bioengineering Institute, China).

### Transmission electron microscope (TEM)

After centrifugation, cells were fixed in 2.5% glutaraldehyde for 2 h and post-fixed with 1% osmium tetroxide for 1 h. Samples were then dehydrated and embedded, and ultrathin sections were imaged using a TEM.

### Immunofluorescence (IF) detection

Cells were fixed with paraformaldehyde, permeabilized with Triton, and blocked with goat serum. After overnight incubation with primary antibodies at 4 °C, samples were treated with fluorescent secondary antibodies (1 h, room temperature). Nuclei were counterstained with DAPI (Cat No. C1006, Beyotime, China), and images were acquired using a microscope.

### Fluorescence in situ hybridization (FISH)

All FISH probes (tRF-Ser, U6, and 18S probes) were synthesized by GenePharma (Shanghai, China), and experiments were conducted using a Cell Fluorescence in Situ Hybridization Kit (Cat No. F12101, GenePharma, China). Briefly, probes were hybridized with cells to localize the subcellular distribution of target molecules via fluorescent labeling, and nuclei were counterstained with DAPI. Fluorescence signals were detected using a microscope, with probe sequences detailed in Supplementary Table 3.

### The nuclear and cytoplasmic protein extraction assay

The cytoplasmic protein and nuclear protein extraction experiment utilized a nuclear and cytoplasmic protein extraction kit (Cat No. PK10014, Proteintech). In brief, following the manufacturer’s protocol, proteins were isolated from the cell supernatant and analyzed via WB assay.

### Dual luciferase reporter gene assay

The HSPA8 promoter region was cloned into the pGL4.10 vector to generate wild-type (WT) and mutant (MT) reporter constructs (Abiotech; sequences in Supplementary Table 4). Luciferase assays were performed using the Dual-Luciferase Reporter Assay System (Cat No. N1610, Promega, USA), and relative luciferase activity was calculated by normalizing firefly luciferase to renilla luciferase.

### RNA pull-down assay and mass spectrometry analysis

For the RNA pull-down assay, biotin-labeled tRF-Ser and its antisense probes (synthesized by GenePharma, Supplementary Table 5) were used. Bound proteins were detected using a Fast Silver Stain Kit (Cat No. P0017S, Beyotime), followed by mass spectrometry analyses conducted at the Advanced Medical Research Institute of Shandong University (Jinan, China).

### RNA immunoprecipitation (RIP)

The RIP assay was conducted using a RIP kit (Cat No. P0101, Geneseed, China). Briefly, cell lysates were incubated with protein A/G magnetic beads (Cat No. HY-K0202, MCE) conjugated to either target antibody or IgG control antibody. Bound RNAs were extracted using Trizol reagent, and target RNA was detected by qRT-PCR.

### Crosslinking immunoprecipitation (CLIP)

The CLIP assay was performed using the CLIP Kit (Cat No. Bes3014, BersinBio, China). Briefly, cells were UV-crosslinked and lysed, followed by the collection of cell lysates for immunoprecipitation. Subsequently, protein digestion and RNA extraction were conducted to enable analysis via CLIP-qPCR.

### Co-immunoprecipitation (Co-IP) assay

Briefly, cell lysates were incubated with target-specific antibody or normal IgG isotype control pre-bound to protein A/G magnetic beads. Immunoprecipitated proteins were analyzed by WB assays, with input lysates as a positive control.

### Chromatin immunoprecipitation (ChIP)

ChIP was performed using a ChIP kit (Cat No. 26157, Thermo Fisher Scientific). Briefly, cell lysates were incubated with target-specific antibody or normal IgG isotype control bound to protein A/G magnetic beads. Precipitated DNA fragments were analyzed by quantitative PCR, with primer sequences provided in Supplementary Table 1.

### Immunohistochemistry (IHC) and hematoxylin-eosin staining (H&E)

Tissue samples were processed for IHC staining and H&E staining using an immunohistochemical kit (PV-9000, Zsgb-Bio, China) and a hematoxylin-eosin staining kit (Cat No. G1120, Solarbio), respectively.

### Animal model

Four-week-old male BALB/c nude mice (Charles River) were maintained under SPF conditions. Three experimental models were established: ① subcutaneous xenograft: mice (*n* = 5 per group) received subcutaneous injections of 1 × 10⁶ MKN-45 cells (100 μL PBS). Tumor volume was measured every 5 days. Mice were euthanized after 4 weeks, and tumor tissues were collected. ② Lung metastasis model: tail vein injection of 1 × 10⁶ MKN-45 cells (100 μL PBS). Metastatic progression was monitored after 4 weeks by an in vivo imaging system (IVIS Lumina Series III, USA), with fluorescence signal quantified using Living Image software. Lung tissues were collected following euthanasia. Then, the quantification of metastatic nodules from H&E-stained lung sections was performed by two researchers who were blinded to the group assignments. ③ Drug intervention model: six days after inoculation, mice bearing OE-Ctrl/OE-tRF-Ser MKN-45 cells were randomized into three groups (*n* = 5/group): a control group (DMSO), an Erastin group (5 mg/kg), and an Erastin (5 mg/kg) + 5-FU (5 mg/kg) group. These tumor-bearing mice were intraperitoneally injected with drugs every 4 days. Tumor size was measured every 5 days, and tumor tissues were collected after 4 weeks.

### Statistical analysis

All quantitative data were expressed as mean ± standard deviation (SD) from at least three independent experiments. Statistical analyses were conducted using GraphPad Prism 8.0 and R software (version 4.5.1). Group comparisons used Student’s t-test or Mann-Whitney U test, as appropriate. Paired t-test was applied to compare tRF-Ser expression in tumor tissues and corresponding normal tissues. The association between tRF-Ser expression and clinicopathological characteristics was analyzed by the chi-square test. Survival analysis employed Kaplan-Meier curves with the log-rank test. A *P* value < 0.05 was considered statistically significant.

## Results

### tRF-Ser is downregulated in GC and correlates with favorable survival

**Fig. 1:**
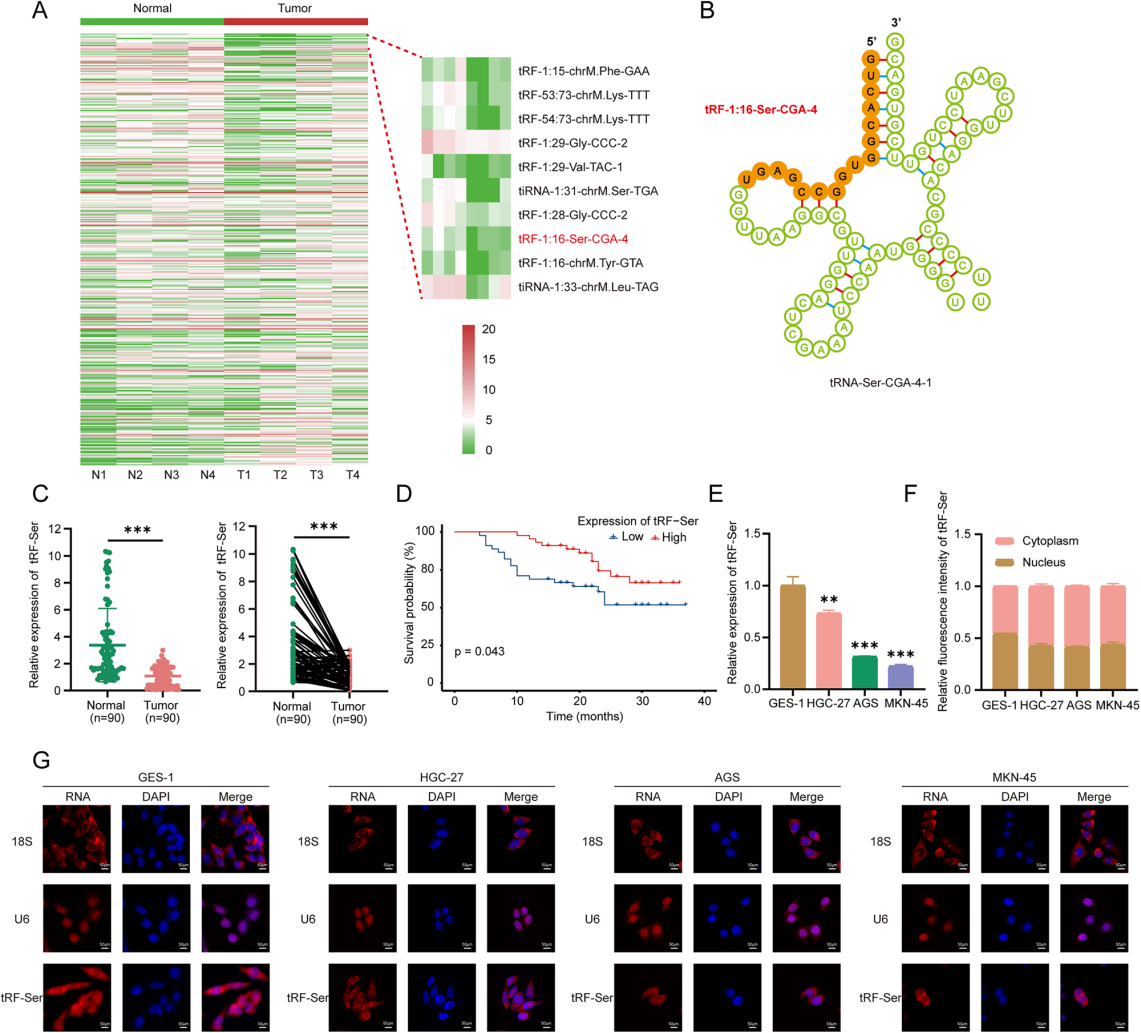
tRF-Ser is downregulated in GC and correlates with favorable prognosis. **A** Heatmaps of tsRNAs expression profiles in four paired GC and adjacent normal tissues. **B** Schematic of sequence feature and genomic location for tRF-1:16-Ser-CGA-4 derived from tRNA-Ser-CGA-4-1. **C** qRT-PCR analyses of tRF-Ser expression in 90 paired GC tissues and adjacent normal tissues. **D** Kaplan-Meier overall survival curves for GC patients stratified by high or low tRF-Ser expression. **E** tRF-Ser expression levels in a normal gastric mucosal epithelial cell line (GES-1) and three GC cell lines (HGC-27, AGS, and MKN-45) as determined by qRT-PCR. **F**, **G** FISH images showed that tRF-Ser was approximately uniformly distributed in the nucleus and cytoplasm of GES-1 cells, whereas in GC cell lines (HGC-27, AGS, and MKN-45), it exhibited a marked reduction in intranuclear signal. Data are expressed as mean ± SD. (Student′s t-test, Mann-Whitney U test, Paired t-test, Log-rank test, **p < 0.01, and ***p < 0.001).

High-throughput sequencing of tRFs/tiRNAs in four paired GC/adjacent tissues identified a novel 5’-derived tsRNA, tRF-1:16-Ser-CGA-4 (designated tRF-Ser; MINTbase ID: tRF-19-16M4PU24; Fig. 1A, B), which had not been previously characterized in GC. tRF-Ser was significantly downregulated in GC tissues by sequencing (Fig. 1A), and a similar result was observed in an independent cohort of 90 GC patients at our center (Fig. 1C). Clinically, low tRF-Ser expression was associated with larger tumor size (≥5 cm), advanced T stage (T3-4), higher lymph node metastasis (N1-3), and advanced TNM stage (III) (Table 1), and predicted poor overall survival (Fig. 1D). Consistent with clinical findings, tRF-Ser was reduced in multiple GC cells (HGC-27, AGS, and MKN-45) compared to normal gastric epithelial cells (GES-1) (Fig. 1E). Subcellular localization revealed predominant nuclear and cytoplasmic distribution in GES-1 cells but markedly reduced nuclear signals in GC cells (Fig. 1F, G), suggesting a potential link between its nuclear distribution and oncogenic function. Taken together, these results identify tRF-Ser as a novel downregulated tsRNA in GC with prognostic and therapeutic potential.

**Table 1:** Correlation between tRF-Ser expression and clinicopathological characteristics in 90 GC patients.

Parameters	Cases	tRF-Ser expression	tRF-Ser expression	*p* value
		Low	High	
**Total**	90	45	45	-
**Age**				0.46
<60	21	9	12	
≥60	69	36	33	
**Gender**				0.82
Male	63	31	32	
Female	27	14	13	
**Tumor size**				<0.001
<5 cm	59	23	36	
≥5 cm	31	22	9	
**Tumor invasion**				0.03
T1-T2	30	10	20	
T3-T4	60	35	25	
**Lymph node metastasis**				0.02
N0	37	13	24	
N1-N3	53	32	21	
**TNM stage**				0.04
I-II	50	20	30	
III	40	25	15	

### tRF-Ser suppresses GC progression in vitro

**Fig. 2:**
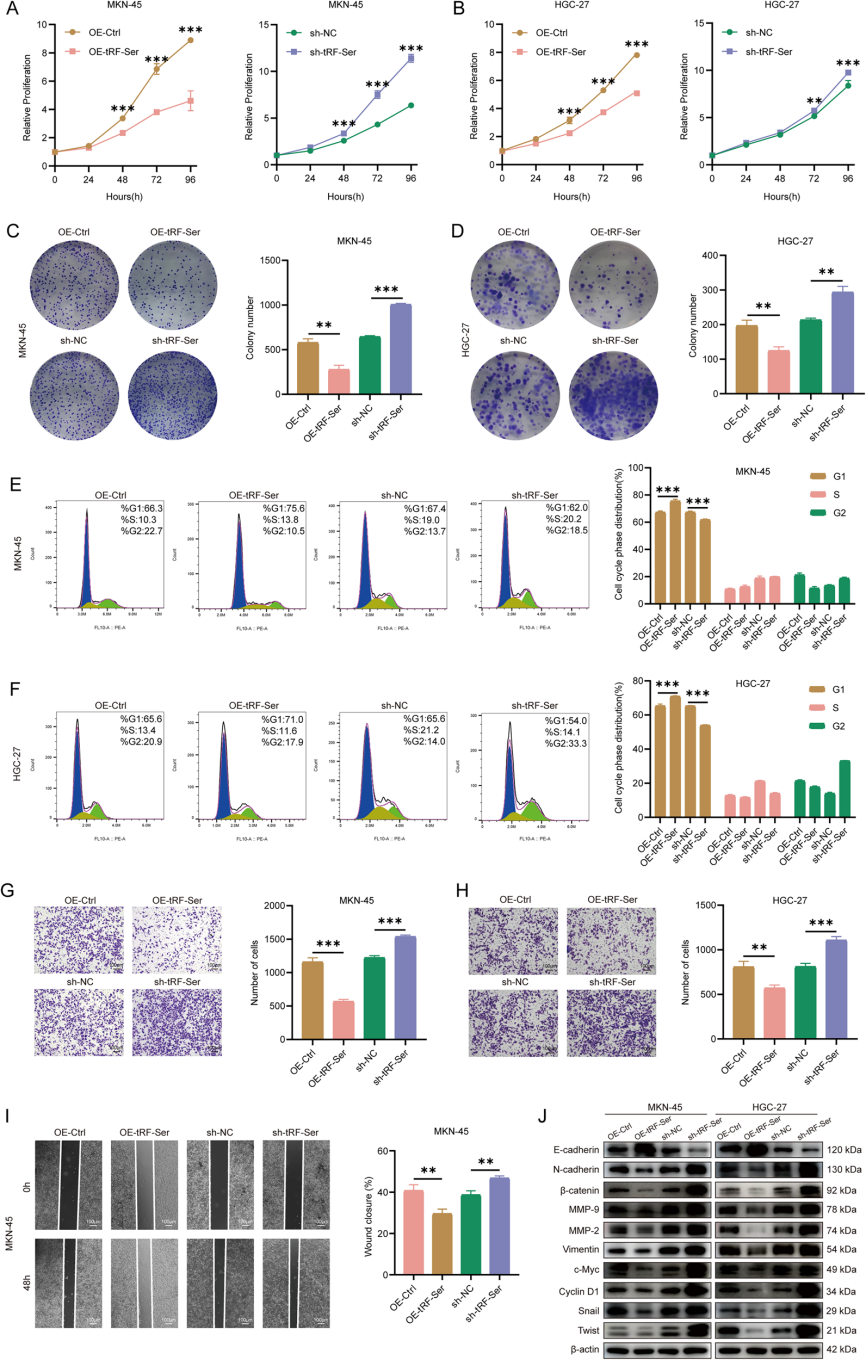
tRF-Ser inhibits GC progression in vitro. **A, B** Cell viability was measured by CCK-8 assays in MKN-45 (**A**) and HGC-27 (**B**) cells following tRF-Ser overexpression or knockdown. **C, D** Colony formation ability was assessed by colony formation assays in MKN-45 (**C**) and HGC-27 (**D**) cells with tRF-Ser overexpression or knockdown. **E, F** Cell cycle distribution was analyzed by flow cytometry assays in MKN-45 (**E**) and HGC-27 (**F**) cells after tRF-Ser overexpression or knockdown. **G, H** Cell invasion was evaluated using Transwell assays in MKN-45 (**G**) and HGC-27 (**H**) cells with tRF-Ser overexpression or knockdown. **I** Cell migration was assessed by wound healing assays in MKN-45 cells with tRF-Ser overexpression or knockdown. **J** WB analyses of key EMT markers in GC cells with modulated tRF-Ser expression. Data are expressed as mean ± SD. (Student′s *t*-test, **p < 0.01, and ***p < 0.001).

To elucidate the biological functions of tRF-Ser in GC progression, we used MKN-45 (the lowest endogenous expression of tRF-Ser) and HGC-27 (highest endogenous expression of tRF-Ser) cell lines for transient transfection with tRF-Ser mimics or inhibitors (Supplementary Fig. 1A, B) and stable lentiviral overexpression or knockdown (Supplementary Fig. 1C, D). Ectopic expression of tRF-Ser significantly inhibited cell proliferation (CCK-8 and colony formation assays; Fig. 2A–D), induced G1 phase cell cycle arrest (Fig. 2E, F), reduced cell invasion and migration (Transwell and wound healing assays; Fig. 2G-I and Supplementary Fig. 1E–G), and suppressed EMT (WB assay; Fig. 2J). Conversely, tRF-Ser knockdown promoted these malignant activities. These results suggest that tRF-Ser may be a critical tumor suppressor in GC.

### tRF-Ser binds to the CNBP protein and regulates its subcellular localization

**Fig. 3:**
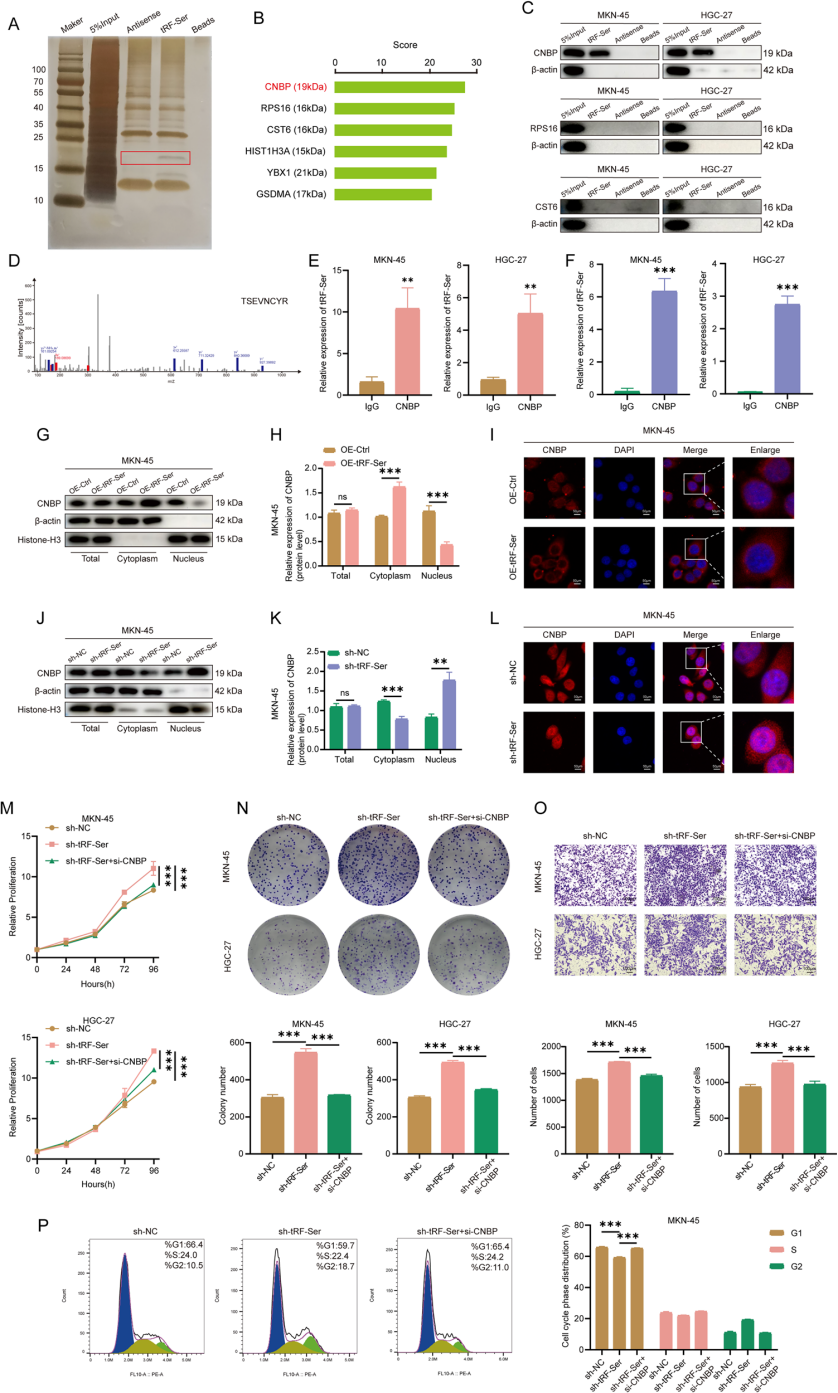
tRF-Ser directly binds to CNBP and restricts its nuclear localization. **A** RNA pull-down combined with silver staining to identify differentially bound proteins with tRF-Ser in MKN-45 cells. **B** List of the top six candidate binding proteins identified from the differential band, ranked by score. **C** Validation of the tRF-Ser-CNBP/RPS16/CST6 interaction by RNA pull-down assays in MKN-45 and HGC-27 cells. **D** The unique peptide of CNBP identified by mass spectrometry assay. **E** Validation of the tRF-Ser-CNBP interaction by RIP assays in MKN-45 and HGC-27 cells. **F** Validation of the tRF-Ser-CNBP interaction by CLIP assays in MKN-45 and HGC-27 cells. **G**–**I** WB (**G**, **H**) and IF (**I**) assays showed tRF-Ser overexpression inhibited CNBP nuclear accumulation in MKN-45 cells. **J**–**L** WB (**J, K**) and IF (**L**) assays showed tRF-Ser knockdown promoted CNBP nuclear accumulation in MKN-45 cells. **M**–**P** CNBP knockdown reversed the pro-tumorigenic effects of tRF-Ser knockdown. Functional assays including CCK-8 (**M**), colony formation (**N**), Transwell invasion (**O**), and cell cycle analysis by flow cytometry (**P**) in GC cells. Data are expressed as mean ± SD. (Student′s *t*-test, **p < 0.01, and ***p < 0.001). ns means no significant.

Studies have indicated that tsRNAs could function by binding to proteins [15, 16]. To uncover the underlying mechanism of tRF-Ser-mediated GC inhibition, we employed RNA pull-down coupled with silver staining and mass spectrometry to identify potential binding partners. Among the proteins corresponding to the differential silver-stained band, CNBP (19 kDa) received the highest matching score (Fig. 3A–D, Supplementary Table 6). Then, to screen for protein that might interact with tRF-Ser, we selected CNBP, RPS16, and CST6 for independent RNA pull-down experiments based on the ranking of candidate protein scores. Notably, only CNBP, a cellular nucleic acid-binding protein, was pulled down by tRF-Ser but not its antisense RNA (Fig. 3C). Furthermore, the specific interaction between tRF-Ser and CNBP was shown by RIP (Fig. 3E) and CLIP assays (Fig. 3F).

Notably, modulating tRF-Ser expression did not affect CNBP mRNA or protein levels, and vice versa (Supplementary Fig. 1H–J). Based on reports that tsRNAs could regulate the subcellular localization of their binding partners [15, 16], we hypothesized that tRF-Ser might function by altering CNBP distribution. Nuclear-cytoplasmic fractionation (Fig. 3G, H, J, K, Supplementary Fig. 1K–N) and IF (Fig. 3I, L, Supplementary Fig. 1O, P) assays validated this hypothesis: tRF-Ser expression was negatively correlated with CNBP nuclear accumulation. Overexpression of tRF-Ser promoted the accumulation of CNBP in the cytoplasm, whereas knockdown of tRF-Ser increased its enrichment in the nucleus.

To functionally interrogate the tRF-Ser/CNBP axis in GC, we generated a CNBP overexpression model and selected si-CNBP-2 (which showed the highest knockdown efficiency; Supplementary Fig. 1Q, R) for rescue experiments. CNBP knockdown effectively reversed the pro-oncogenic effects induced by tRF-Ser knockdown, including reduced proliferation/invasion and aggravated G1 arrest (Fig. 3M–P, Supplementary Fig. 1W). These findings suggest that tRF-Ser may exert its tumor-suppressive effects by inhibiting the nuclear localization of CNBP, revealing the control of protein localization as a core mechanism of the tRF-Ser/CNBP functional axis.

### CNBP promotes GC progression both in vitro and in vivo

As a conserved nucleic acid-binding protein, the transcription factor CNBP is aberrantly overexpressed in multiple malignancies, including pancreatic cancer [23], ovarian cancer [24], and neuroblastoma [25]. To explore its role in GC, we first identified its upregulation. Interrogation of public data (TIMER database; Supplementary Fig. 2A) revealed significant CNBP overexpression in GC, and high CNBP expression was associated with poor OS survival (KM-Plotter; Supplementary Fig. 2B). Then, similar results were validated in our cohort of 30 paired GC tissues by IHC (Supplementary Fig. 2C, D) and qRT-PCR (Supplementary Fig. 2E, F).

We then established lentiviral CNBP overexpression and knockdown models (Supplementary Fig. 1S–V) to functionally characterize CNBP in GC. In vitro, CNBP overexpression enhanced cell proliferation, invasion, migration, cell cycle progression, and EMT, whereas its knockdown suppressed these malignant effects (Supplementary Fig. 2G–O). Furthermore, nuclear-cytoplasmic fractionation assays indicated that CNBP facilitated the nuclear translocation of β-catenin, suggesting activation of the Wnt signaling pathway (Supplementary Fig. 2P). This pro-tumorigenic role was similarly observed in vivo: CNBP overexpression accelerated subcutaneous tumor growth (Supplementary Fig. 2Q–S), while its knockdown significantly reduced tumor burden (Supplementary Fig. 2T–V). Collectively, these results indicate that CNBP may serve as a key oncogenic driver that promotes GC progression.

### tRF-Ser suppresses GC progression by transcriptionally inhibiting HSPA8

**Fig. 4:**
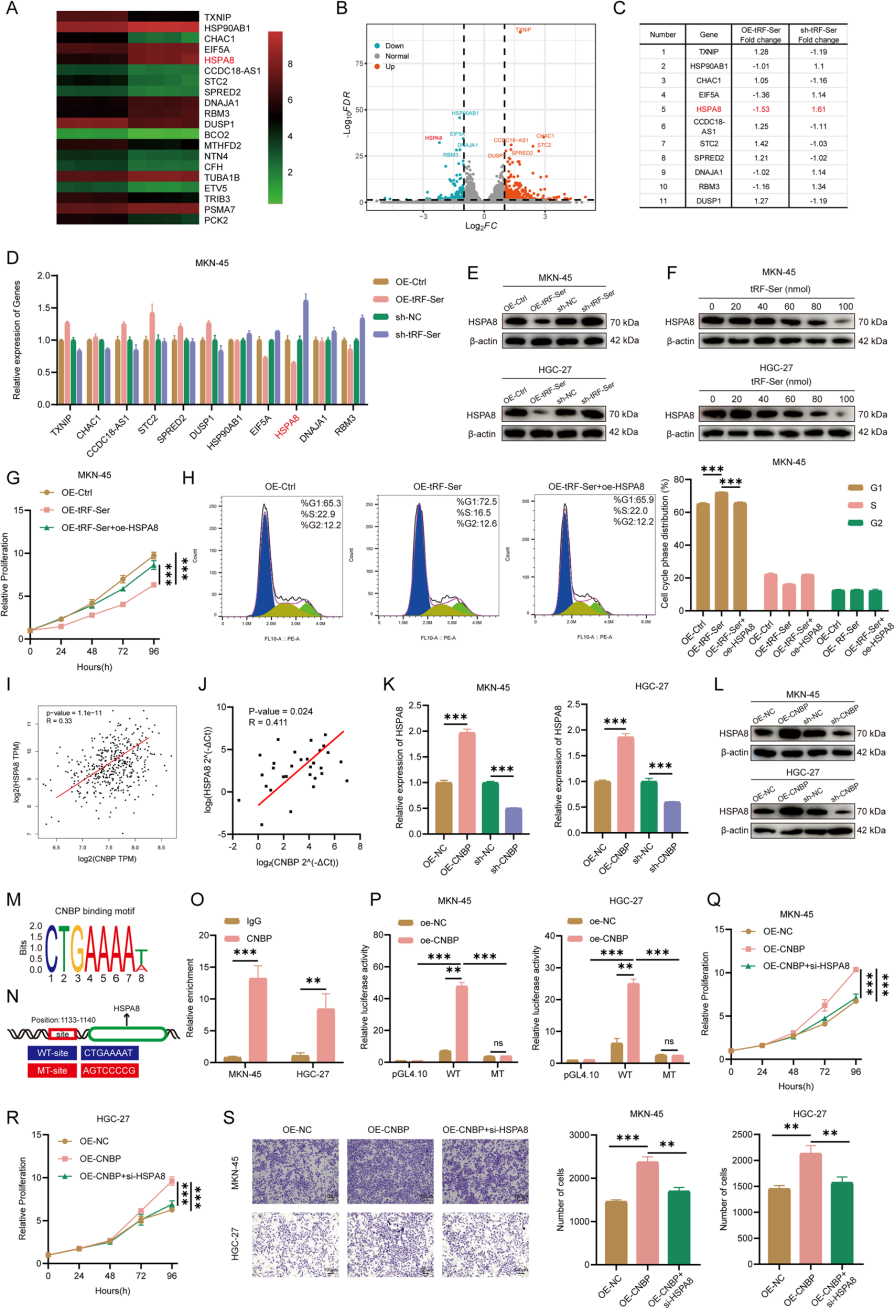
The tRF-Ser/CNBP axis transcriptionally represses HSPA8 to inhibit GC progression. **A, B** Transcriptomic profiling of MKN-45 cells with tRF-Ser overexpression and negative control. Heatmap (**A**) and volcano plot (**B**). **C**, **D** qRT-PCR validation of the top 11 candidate downstream genes in MKN-45 cells with tRF-Ser overexpression and knockdown. **E** WB analyses of HSPA8 protein expression following tRF-Ser modulation in GC cells. **F** Dose-dependent suppression of HSPA8 expression by increasing concentrations of tRF-Ser mimics in GC cells, as shown by WB. **G, H** Rescue experiments showed that HSPA8 overexpression attenuated the anti-proliferative (CCK-8, **G**) and cell cycle-arresting (flow cytometry, **H**) effects of tRF-Ser overexpression in MKN-45 cells. **I, J** Positive correlation between CNBP and HSPA8 expression in GC from public database (GEPIA, **I**) and our cohort of 30 patients (**J**). **K, L** qRT-PCR (**K**) and WB (**L**) analyses of HSPA8 expression upon CNBP modulation in GC cells. **M** Motif sequence specifically recognized by CNBP. **N** The one binding site of CNBP to the promoter region of HSPA8 and the point mutations specifically designed to disrupt the predicted CNBP binding motif (WT-site and MT-site) were shown. **O** ChIP-qPCR analyses showing CNBP enrichment on the HSPA8 promoter in GC cells. **P** Dual-luciferase reporter assays indicating CNBP-mediated activation of the HSPA8 promoter in GC cells. **Q-S** Rescue experiments suggested that HSPA8 knockdown reversed the pro-proliferative (CCK-8, **Q**, **R**) and pro-invasive (Transwell, **S**) effects of CNBP overexpression in GC cells. Data are expressed as mean ± SD. (Student′s *t*-test, **p < 0.01, and ***p < 0.001). ns means no significant.

To further explore the molecular mechanism of tRF-Ser, we performed RNA sequencing in MKN-45 cells with tRF-Ser overexpression and control treatment. The heat map (Fig. 4A), volcano map (Fig. 4B), and qRT-PCR analyses (Fig. 4C, D, Supplementary Fig. 3A, B) showed that HSPA8 was the most significantly regulated target gene among the top 11 candidates, and WB analyses (Fig. 4E, F) also showed that tRF-Ser negatively regulated HSPA8 expression. HSPA8, a gene widely discussed in the literature, has established roles in carcinogenesis [26], ferroptosis [26], protein folding [27], autophagy [28], and necroptosis [29]. However, its specific function remained unclear in GC. Therefore, we investigated HSPA8 as a key downstream effector of tRF-Ser.

First, we found that HSPA8 was significantly upregulated in GC tissues compared to adjacent normal tissues in our cohort of 30 patients, as shown by qPCR and IHC (Supplementary Fig. 3C–F). To investigate if HSPA8 was necessary for the effects of tRF-Ser, we constructed HSPA8 overexpression and knockdown models (Supplementary Fig. 3G–I) and conducted rescue experiments. Overexpression of HSPA8 effectively reversed the tumor-suppressive effects of tRF-Ser overexpression, restoring capabilities in proliferation, invasion, and cell cycle progression (Fig. 4G, H, Supplementary Fig. 3J–O). These findings suggest that tRF-Ser may inhibit GC progression by targeting HSPA8.

### CNBP targets HSPA8 mRNA to promote GC progression

As an important transcription factor, CNBP promotes tumor development by directly binding to promoter regions or G-rich sequences to activate key oncogenes [30]. Furthermore, the nuclear localization signal could further enhance its transcriptional activity [31, 32]. Our study indicated that tRF-Ser inhibited GC progression by accumulating CNBP in the cytoplasm, thereby preventing its nuclear function. Given that our RNA-seq data indicated that tRF-Ser transcriptionally repressed HSPA8, we hypothesized that this effect was mediated by inhibiting CNBP nuclear localization.

Several lines of evidence supported this mechanistic link. First, bioinformatic analysis (GEPIA; Fig. 4I) and our data (Fig. 4J) showed a significant positive correlation between CNBP and HSPA8 expression. Second, modulating CNBP expression (via overexpression or knockdown) correspondingly altered HSPA8 mRNA and protein levels (Fig. 4K, L). Third, a previous study [32] indicated CNBP acted as a transcription factor, recognizing characteristic motif such as CTGAAAAt(a) in the DNA promoter region to activate downstream target genes, and NCBI database analysis revealed the HSPA8 promoter region (position: 1133–1140, sequence: CTGAAAAT) had a sequence that highly matched the CNBP recognition motif (Fig. 4M, N). This interaction was functionally validated: ChIP-qPCR found CNBP enrichment in the HSPA8 promoter (Fig. 4O), and the dual-luciferase reporter assays showed that CNBP overexpression enhanced WT luciferase activity, with blunted activation in MT (CTGAAAAT → AGTCCCCG) (Fig. 4N, P). Finally, rescue experiments showed the functional necessity of HSPA8 in the CNBP pathway. Knockdown of HSPA8 effectively reversed the pro-tumorigenic effects of CNBP overexpression, resulting in inhibition of proliferation and invasion and induction of cell cycle arrest (Fig. 4Q–S, Supplementary Fig. 3P–R). Collectively, these results indicate that CNBP transcriptionally activates HSPA8 in GC by specifically binding to the ‘CTGAAAAT’ core motif in its promoter region.

### The tRF-Ser/CNBP/HSPA8 axis suppresses GC progression

**Fig. 5:**
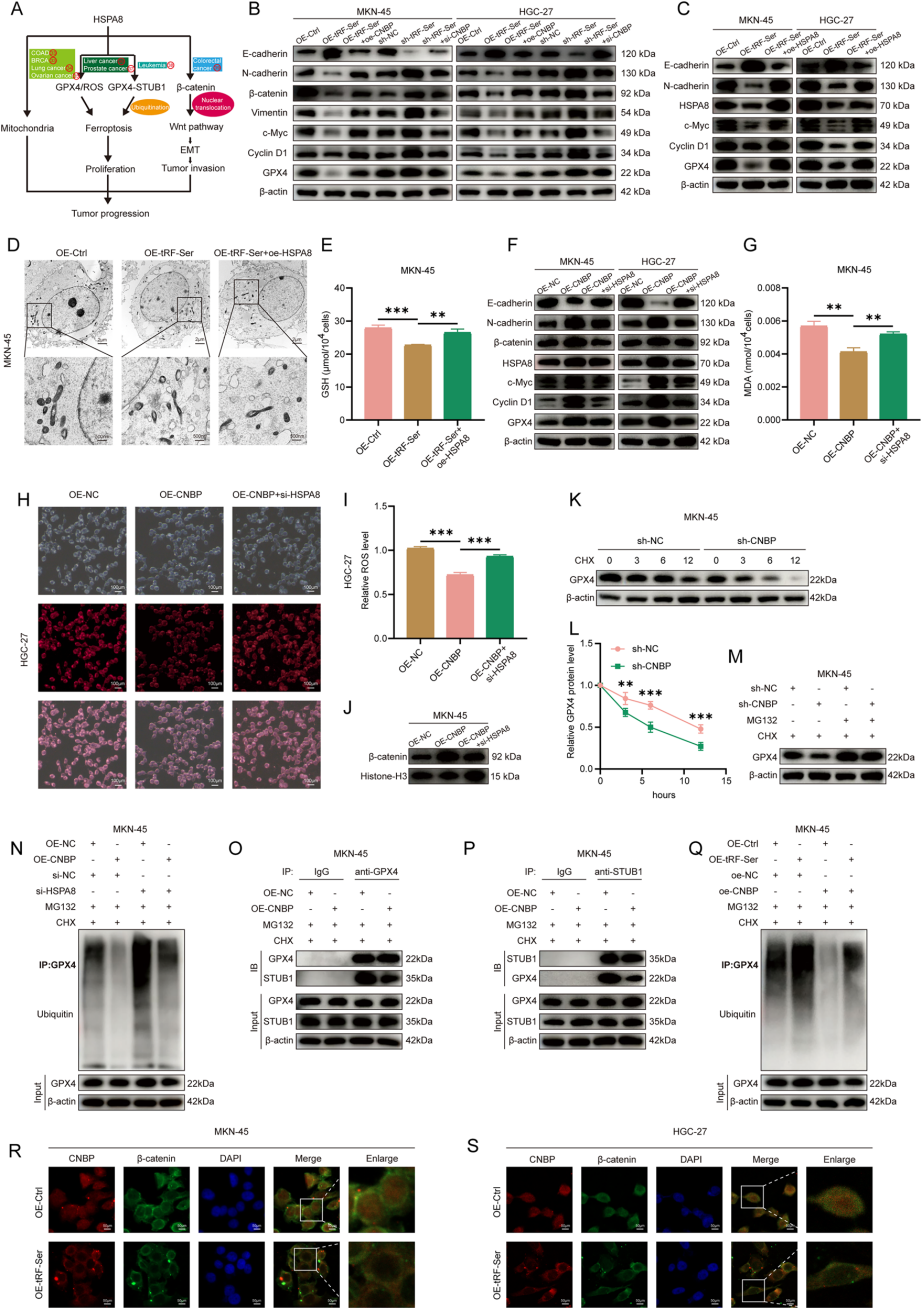
The tRF-Ser/CNBP/HSPA8 signaling axis suppresses GC progression. **A** Schematic diagram summarizing the reported mechanisms of HSPA8 in cancers (references 33-39). **B** WB analyses showed that altering CNBP expression reversed the expression of EMT and ferroptosis markers changed by tRF-Ser. **C** WB analyses indicated that HSPA8 overexpression reversed the changes in protein markers induced by tRF-Ser overexpression. **D** Representative TEM images showed that HSPA8 overexpression reversed the mitochondrial damage induced by tRF-Ser overexpression under Erastin treatment (10 μM). **E** GSH assay showed that HSPA8 overexpression reversed the GSH depletion caused by tRF-Ser overexpression. **F** WB analyses showed that HSPA8 knockdown reversed the expression of protein markers induced by CNBP overexpression. **G**–**I** HSPA8 knockdown reversed the ferroptosis-suppressive effects of CNBP overexpression: MDA levels (**G**) and ROS levels after Erastin induction (4 uM) (**H**, **I**). **J** WB analyses showed that HSPA8 knockdown reversed the β-catenin nuclear translocation promoted by CNBP overexpression. **K**, **L** WB analyses showed that CNBP knockdown reduced GPX4 protein stability (CHX, 50 μg/mL) (**K**), and after CNBP was knocked down, the half-life of GPX4 was approximately reduced from 12 hours to 6 hours (**L**). **M** WB analyses showed that the MG132 (10 μM) reversed the reduction of GPX4 levels induced by CNBP knockdown. **N** WB analysis of GPX4 ubiquitination showed that HSPA8 knockdown reversed the ubiquitination suppressed by CNBP overexpression. **O**, **P** The bidirectional Co-IP assays showed that CNBP overexpression disrupted the STUB1-GPX4 interaction. **Q**. GPX4 ubiquitination assay showed that CNBP overexpression reduced the ubiquitination promoted by tRF-Ser overexpression. **R**, **S** IF assays indicated that tRF-Ser overexpression inhibited nuclear translocation of both CNBP and β-catenin in MKN-45 (**R**) and HGC-27 (**S**) cells. Data are expressed as mean ± SD. (Student′s *t*-test, **p < 0.01, and ***p < 0.001).

As a key gene regulating tumor progression, HSPA8 has been shown to drive malignant tumors through multiple mechanisms (Fig. 5A). In colon cancer [33], breast cancer [33], lung cancer [34], and ovarian cancer [35], it promotes tumor progression by regulating mitochondrial function. In liver cancer [36] and prostate cancer [37], it exerts its effects by modulating oxidative stress markers such as GPX4 or ROS. In leukemia [38], it regulates STUB1-mediated ubiquitination degradation of GPX4, thereby influencing ferroptosis. Additionally, in colorectal cancer [39], HSPA8 facilitates the translocation of β-catenin into the nucleus to activate the Wnt signaling pathway, driving the EMT process. After discovering the critical roles of the tRF-Ser/CNBP, tRF-Ser/HSPA8, and CNBP/HSPA8 axes in GC, we hypothesized that the integrated tRF-Ser/CNBP/HSPA8 axis may inhibit GC progression by inducing ferroptosis and suppressing the Wnt/β-catenin pathway.

Functional assays indicated that tRF-Ser may promote ferroptosis. Under Erastin treatment, tRF-Ser overexpression enhanced cell death, whereas tRF‑Ser knockdown inhibited cell death (CCK-8; Supplementary Fig. 4A, B). Oxidative stress markers (Supplementary Fig. 4C–H) revealed overexpression cells increased ROS and MDA and reduced GSH, with opposite trends in knockdown cells. TEM assays (Supplementary Fig. 4I) showed that under Erastin induction, tRF-Ser overexpressing cells induced pronounced mitochondrial damage (reduced/disappeared cristae, outer membrane rupture, and volume shrinkage), whereas tRF-Ser knockdown exhibited reduced mitochondrial structural damage in MKN-45 cells. WB (Supplementary Fig. 4J) revealed that tRF-Ser downregulated GPX4. Furthermore, we further explored the association between tRF-Ser and ferroptosis. Under the treatment of the ferroptosis inhibitor (ferrostatin-1), the results showed that ferrostatin-1 effectively reversed the pro-ferroptosis effects of tRF-Ser overexpression. Specifically, it restored the protein level of GPX4 that was inhibited by tRF-Ser overexpression (Supplementary Fig. 4K); weakened the promotion of ROS induced by tRF-Ser overexpression (Supplementary Fig. 4L–O); and counteracted the increase in MDA and the decrease in GSH caused by tRF-Ser overexpression (Supplementary Fig. 4P, Q).

Then, we found that CNBP suppressed ferroptosis, as its overexpression attenuated Erastin-induced ROS accumulation and mitochondrial damage relative to knockdown. And CNBP enhanced GPX4 levels and GSH levels and reduced MDA concentrations (Supplementary Fig. 2O, Supplementary Fig. 5A–F). Furthermore, the ferroptosis-inducing effects triggered by CNBP knockdown were effectively counteracted by ferrostatin-1. It restored the downregulated GPX4 protein expression, reversed the elevated ROS/MDA levels and the depleted GSH levels induced by CNBP knockdown (Supplementary Fig. 5G–M).

In the rescue experiments, CNBP knockdown reversed the effects of tRF-Ser knockdown, inhibiting EMT and promoting ferroptosis (Fig. 5B, Supplementary Fig. 6A–G). Further, HSPA8 overexpression reversed the tumor-suppressive effects (inhibition of EMT and induction of ferroptosis) caused by tRF-Ser overexpression (Fig. 5C–E, Supplementary Fig. 6H–M). Then, HSPA8 knockdown blocked the pro-tumor effects of CNBP overexpression, inhibiting β-catenin nuclear translocation and EMT while promoting ferroptosis (Fig. 5F–J, Supplementary Fig. 7A-F).

It has been reported that STUB1, a novel E3 ubiquitin ligase, could promote the ubiquitinatin degradation of GPX4 by interacting with GPX4, thereby inducing ferroptosis and inhibiting the malignant progression of gastrointestinal stromal tumor or leukemia [38, 40]. In addition, another study has indicated that HSPA8 is known to disrupt STUB1-GPX4 binding, blocking GPX4 ubiquitination [38]. Here, we speculated that CNBP may regulate GPX4 stability through the HSPA8-mediated ubiquitination degradation pathway, and our results showed that CNBP knockdown shortened GPX4 protein half-life (the half-life of GPX4 was approximately reduced from 12 hours to 6 hours), an effect reversed by the proteasome inhibitor MG132 (Fig. 5K–M). CNBP overexpression inhibited GPX4 ubiquitination, which was reversed by HSPA8 knockdown (Fig. 5N), whereas CNBP knockdown/HSPA8 overexpression exerted the opposite effect (Supplementary Fig. 7G). Further, bidirectional Co-IP assays revealed that CNBP overexpression disrupted the binding between the STUB1 and GPX4 (Fig. 5O, P). This reveals a mechanism whereby CNBP, via upregulating HSPA8, disrupts STUB1-GPX4 interaction to stabilize GPX4 and inhibit ferroptosis.

Finally, we validated the entire tRF-Ser/CNBP/HSPA8 regulatory axis. Ubiquitination assays showed that CNBP overexpression reversed tRF-Ser-induced GPX4 ubiquitination (Fig. 5Q), while CNBP knockdown antagonized the effect of tRF-Ser knockdown (Supplementary Fig. 7H). IF assays found that tRF-Ser overexpression inhibited the nuclear accumulation of CNBP and β-catenin (Fig. 5R, S).

### tRF-Ser suppresses GC progression in vivo

**Fig. 6:**
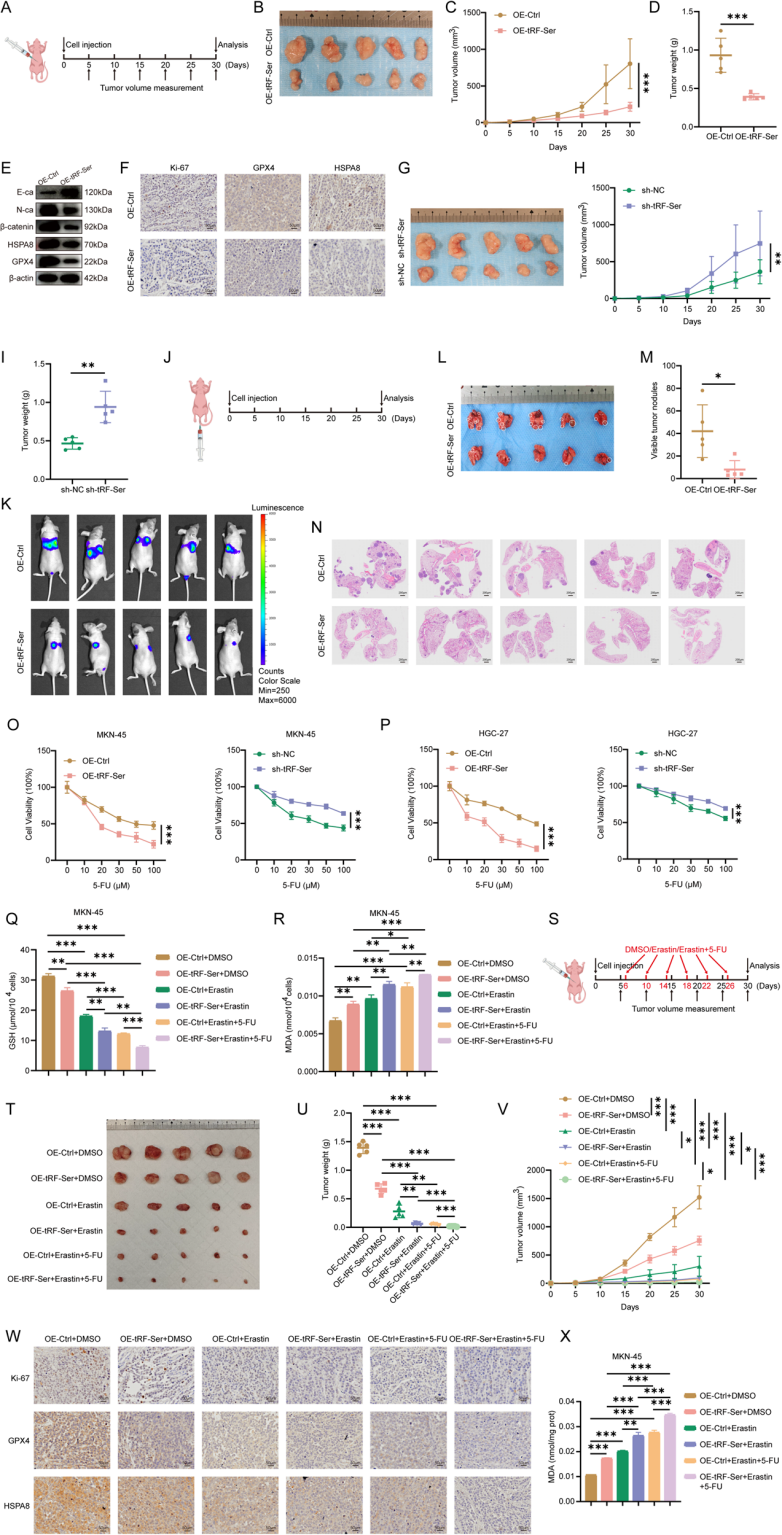
tRF-Ser suppresses tumor growth and metastasis in vivo and enhances 5-FU sensitivity in GC. **A** The workflow of subcutaneous tumorigenesis. **B**–**F** Effects of tRF-Ser overexpression on subcutaneous tumor growth in MKN-45 cells (*n* = 5 mice per group). Representative tumor image (**B**), tumor growth curves (**C**), tumor weights (**D**), WB analyses of protein expression in tumors (**E**), and IHC assays of Ki-67, GPX4, and HSPA8 in tumor sections (**F**). **G**–**I** Effects of tRF-Ser knockdown on subcutaneous tumor growth in MKN-45 cells (*n* = 5). Representative tumor image (**G**), tumor growth curves (**H**), and tumor weights (**I**). **J** The workflow of the lung metastasis model was established via tail vein injection. **K**–**N** Effects of tRF-Ser overexpression on lung metastasis in MKN-45 cells (*n* = 5). In vivo imaging and quantification (**K**), number of lung metastatic nodules (**L**–**N**), and H&E staining of lung tissues (**N**). **O, P** Cell viability (CCK-8 assay) in MKN-45 (**O**) and HGC-27 (**P**) cells with tRF-Ser modulation after 5-FU treatment. **Q, R** Effects of tRF-Ser modulation on GSH (**Q**) and MDA (**R**) levels in MKN-45 cells treated with 5-FU (20 μM) + Erastin (10 μM). **S** The workflow for the combination therapy experiment in subcutaneous xenografts. **T**–**X** Effects of combination therapy (Erastin (5 mg/kg) + 5-FU (5 mg/kg)) in mice bearing tRF-Ser-overexpressing MKN-45 cell tumors (*n* = 5). Representative tumor image (**T**), tumor weights (**U**), tumor growth curves (**V**), IHC analyses (**W**) showing significantly reduced levels of Ki-67, GPX4, and HSPA8 in the combination treatment group, and MDA experiment (**X**) showed that the drug intervention could significantly increase the MDA levels of tumor tissues. Data are expressed as mean ± SD. (Student′s *t*-test, *p < 0.05, **p < 0.01, and ***p < 0.001).

In vivo functional studies using two distinct models indicated that tRF-Ser exerted its tumor-suppressive effects. First, in subcutaneous xenografts, tRF-Ser overexpression significantly impaired tumor growth, reducing both tumor volume and weight (Fig. 6A–D [41]), while its knockdown enhanced tumorigenesis (Fig. 6G–I). Molecular analyses of these xenografts by WB and IHC showed the downregulation of EMT markers, GPX4, and HSPA8 in the tRF-Ser overexpression group (Fig. 6E, F). Second, in lung metastasis model, tRF-Ser overexpression dramatically reduced the metastatic burden in lungs, manifested as a decrease in both the number and size of metastatic nodules (Fig. 6J–N). Together, these findings suggest that tRF-Ser may act as a suppressor of GC progression and metastasis in vivo.

### tRF-Ser positively enhances 5-FU sensitivity in GC cells

Tumorigenesis and progression are driven by the dysregulation of multiple cellular pathways, with aberrations in cell death and chemoresistance being pivotal determinants of cancer malignancy and treatment failure [4, 17]. Recent studies have implicated tRFs in these processes; for instance, tRF-23-Q99P9P9NDD modulates ferroptosis in GC [42], and tRF-27 increases trastuzumab tolerance in HER2+ breast cancer [43]. Given the known role of tRF-Ser in suppressing GC by inhibiting EMT and promoting ferroptosis, we investigated whether it also enhanced chemosensitivity to 5-FU by perturbing energy metabolism.

**Fig. 7:**
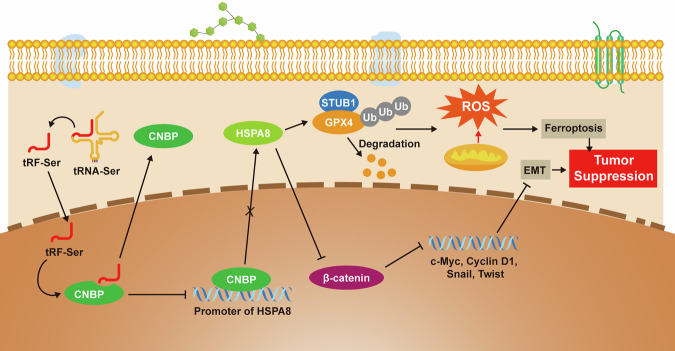
A mechanism schematic diagram of tRF-Ser-mediated tumor suppression in GC. tRF-Ser binds to the transcription factor CNBP and promotes its accumulation in the cytoplasm, thereby repressing CNBP-mediated transcriptional activation of HSPA8. Then, the tRF-Ser/CNBP/HSPA8 axis suppresses β-catenin-mediated EMT and induces GPX4 ubiquitination-dependent ferroptosis to inhibit GC progression.

Functional assays indicated that tRF-Ser sensitized GC cells to 5-FU. Under 5-FU treatment, cell viability (CCK-8 assay) was significantly lower in tRF-Ser-overexpressing cells, but higher in tRF-Ser knockdown cells (Fig. 6O, P). We then evaluated a novel combination strategy: tRF-Ser overexpression combined with Erastin and 5-FU. In vitro, this triple combination synergistically increased MDA levels and depleted GSH more effectively than any single or dual treatment (Fig. 6Q, R, Supplementary Fig. 7I, J). In vivo, it produced the most pronounced anti-tumor effect, markedly reducing subcutaneous tumor volume and weight compared to all other groups (Fig. 6S–V). Histological and biochemical analyses of these tumors revealed significantly lower expression of Ki-67, GPX4, and HSPA8, coupled with higher MDA levels, in the triple-therapy group (Fig. 6W, X). In summary, our findings suggest that tRF-Ser may inhibit GC progression through a multi-pronged function: suppressing EMT, inducing ferroptosis, and sensitizing tumors to 5-FU chemotherapy. This tumor-suppressive function is mechanistically dependent on the tRF-Ser/CNBP/HSPA8 signaling axis (Fig. 7).

## Discussion

GC remains a common malignancy with a poor prognosis, where therapeutic efficacy is often hampered by tumor progression and chemoresistance, highlighting the critical need for novel molecular targets. This study identifies tRF-Ser, a previously uncharacterized tsRNA that is significantly downregulated in GC. We show that tRF-Ser may act as a tumor suppressor by orchestrating multi-synergistic anti-tumor programs: inhibiting EMT, activating ferroptosis, and sensitizing cancer cells to 5-FU chemotherapy. Mechanistically, tRF-Ser may execute its function by directly binding to the transcription factor CNBP, thereby affecting its nuclear localization and subsequent binding to the HSPA8 promoter to repress HSPA8 transcription. The tRF-Ser/CNBP/HSPA8 axis may function through two distinct downstream pathways, such as inhibiting EMT by suppressing β-catenin nuclear translocation and driving ferroptosis by promoting STUB1-mediated ubiquitination degradation of GPX4. These findings not only unveil a novel tRF-Ser-mediated metabolic-energy signaling axis in GC but also provide a theoretical foundation for developing tRF-Ser-based targeted therapeutic strategies.

Ferroptosis is a key cell death pathway triggered by oxidative stress, and dysregulation of ferroptosis is often closely associated with chemoresistance and treatment failure [17, 19]. To counter this, combining standard chemotherapy with ferroptosis represents a transformative therapeutic approach. Evidence supports this across malignancies: in GC, miR-522 inhibits ferroptosis by targeting ALOX15 and blocking ROS accumulation, thereby affecting tumor cell sensitivity to cisplatin and paclitaxel [44]; in esophageal cancer, PLK1 knockdown reduces NADPH and GSH levels by inhibiting the pentose phosphate pathway, thereby promoting ferroptosis and improving chemoradiotherapy sensitivity [45]; in pancreatic cancer, HSPA5 blocks ferroptosis by inhibiting GPX4 protein degradation and lipid peroxidation, affecting sensitivity to gemcitabine [46]. Echoing this paradigm, we show that tRF-Ser may exert its tumor-suppressive effects in GC through a dual mechanism: potentiation of ferroptosis and sensitization to 5-FU. These results reveal tRF-Ser-targeted therapy as a promising strategy to resensitize GC tumors to conventional chemotherapy.

The subcellular localization diversity of sncRNAs forms the basis for their precise regulation of gene functions [47, 48]. tsRNAs, a novel characterized class of sncRNAs, contribute to gene regulatory networks through mRNA silencing, translational regulation, epigenetic control, intercellular communication, and protein interactions [8, 15, 16]. Here, we found that tRF-Ser was markedly depleted in the nuclei of GC cells, implicating the loss of its nuclear activity in malignant progression. We mechanistically linked this observation to a direct interaction between tRF-Ser and the nucleic acid-binding protein CNBP, which influenced the nuclear localization of CNBP. Through a combination of transcriptomic profiling and functional validation, we further identified HSPA8 as a key downstream effector and found the significance of both the tRF-Ser/CNBP and tRF-Ser/HSPA8 axes in GC. These data collectively support a hypothesis that tRF-Ser fine-tuned HSPA8 transcription by modulating the activity of transcriptional factor CNBP.

CNBP is a conserved nucleic acid-binding protein and transcription factor that promotes oncogenesis by activating downstream genes through binding to specific promoter elements (e.g., CTGAAAAt(a)) or G-rich sequences [30–32, 49]. Its activity is enhanced by nuclear localization [31, 32], as demonstrated in cancers like melanoma and lung cancer, where it transcriptionally upregulates pro-metastatic factors (MMP-2, MMP-14, E2F2) [31]. Building on this established role, our study delineates a novel regulatory circuit in GC. We find that the tRF-Ser may function as a critical upstream regulator by promoting the accumulation of CNBP in the cytoplasm, thereby limiting its nuclear function. Furthermore, we identify HSPA8 as a direct transcriptional target of CNBP and show the CNBP/HSPA8 axis as a pivotal pro-tumorigenic pathway in GC. Thus, our work not only corroborates the importance of CNBP subcellular localization but also shows a complete signaling module from non-coding RNA to transcription factor to effector.

The molecular chaperone HSPA8, a core HSP70 family member, is a well-established mediator of cellular homeostasis whose dysregulation is oncogenic across diverse cancers. It promotes tumor progression through context-specific mechanisms: driving Wnt signaling via β-catenin nuclear import in colorectal cancer [39]; inhibiting ferroptosis by stabilizing GPX4 in leukemia [38]; modulating redox homeostasis in liver and prostate cancers [36, 37]; and affecting mitochondrial function in lung and ovarian cancers [34, 35]. Despite its recognized role in other malignancies, the function of HSPA8 in GC remains unexplored. Here, we not only uncover its oncogenic activity in GC but also integrate it into a novel regulatory pathway. We indicate that the tRF-Ser/CNBP/HSPA8 axis constrains GC progression via two coordinated mechanisms: inhibiting EMT by promoting β-catenin accumulation in the cytoplasm and enhancing ferroptosis via ubiquitination degradation of GPX4.

However, this study has several limitations. First, the precise domains mediating the tRF-Ser-CNBP interaction—a critical determinant of its regulatory specificity—remain to be mapped. Second, although CNBP nucleocytoplasmic shuttling is known to be regulated by post-translational modification (PTM) like phosphorylation [31, 50], whether tRF-Ser controls CNBP localization by altering its PTM status remains an open question. Future work will therefore be dedicated to characterizing the structural basis of this RNA-protein interaction and investigating the potential of tRF-Ser to regulate CNBP function through PTM.

## Conclusions

In conclusion, our work unveils a previously unrecognized tsRNA-regulated signaling network (tRF-Ser/CNBP/HSPA8) that integrates metabolic cell death (ferroptosis) with cellular differentiation (EMT) and drug response. These findings not only deepen our understanding of GC pathogenesis but also provide a strong rationale for developing tRF-Ser-based therapeutics and biomarkers for GC management.

## Supplementary information

Supplementary Information

Supplementary Figure 1

Supplementary Figure 2

Supplementary Figure 3

Supplementary Figure 4

Supplementary Figure 5

Supplementary Figure 6

Supplementary Figure 7

Supplementary Figure 8

## Data Availability

The data that support the findings of this study are available from the corresponding author upon reasonable request.
