# Supplementary material for: A new candidate tumor suppressor tRF-Ser inhibits gastric cancer progression by regulating the CNBP/HSPA8 axis
Source: Cell Death Dis. 2026 Mar 25;17(1):379. doi: 10.1038/s41419-026-08608-1 (PMC13039117; doi:10.1038/s41419-026-08608-1)
Supplement: Supplementary file 1 — Supplementary Information [file 41419_2026_8608_MOESM1_ESM.docx]

**Supplementary Information**

**Supplementary Fig 1. A-B.** Transfection efficiency of tRF-Ser mimics and inhibitors in GC cells, determined by qRT-PCR. **C-D.** Transfection efficiency of lentiviruses for tRF-Ser overexpression and knockdown in GC cells, assessed by qRT-PCR. **E-F.** Transwell migration assays in GC cells with tRF-Ser modulation. **G.** Wound healing assays for cell migration in HGC-27 cells with tRF-Ser modulation. **H-I.** tRF-Ser modulation did not affect CNBP expression. qRT-PCR (**H**) and WB (**I**) analyses. **J.**CNBP modulation did not affect tRF-Ser expression, as shown by qRT-PCR. **K-P.** WB (**K, M**) and IF (**O**) assays showed tRF-Ser overexpression inhibited CNBP nuclear accumulation in HGC-27 cells. WB (**L, N**) and IF (**P**) assays showed tRF-Ser knockdown promoted CNBP nuclear accumulation in HGC-27 cells. **Q-R.**Validation of CNBP overexpression plasmid and siRNAs transfection efficiency in MKN-45 (**Q**) and HGC-27 (**R)**cells by qRT-PCR. **S-T.** Validation of lentiviral-mediated CNBP overexpression and knockdown (using si-CNBP-2) in MKN-45 (**S**) and HGC-27 (**T**) cells by qRT-PCR. **U-V.** Validation of CNBP protein expression upon lentiviral modulation in MKN-45 (**U**) and HGC-27 (**V**) cells by WB. **W.** Cell cycle analysis by flow cytometry assays in HGC-27 (**W**) cells showed that CNBP knockdown reversed the cell cycle process induced by tRF-Ser knockdown. Data are expressed as mean ± SD. (Student′s t-test, *, p < 0.05, **, p < 0.01, and ***, p < 0.001). ns means no significant.

**Supplementary Fig 2. A.** Expression levels of the CNBP gene in different TCGA tumors via the Timer database. **B.** Kaplan-Meier overall survival analysis of GC patients stratified by high or low CNBP expression (KM-Plotter database). **C-F.** IHC (**C, D**) and qRT-PCR (**E, F**) assays of 30 pairs of GC and adjacent tissues showing high CNBP expression in GC. **G-V.** Functional characterizations of CNBP in GC. In vitro (**G-P**): Cell proliferation (CCK-8 assay) in MKN-45 (**G**) and HGC-27 (**H**) cells upon CNBP modulation. Cell cycle distribution (flow cytometry) in MKN-45 (**I**) and HGC-27 (**J**) cells. Cell invasion (**K, L**) and migration (**M, N**) (Transwell assay) in MKN-45 and HGC-27 cells. WB analyses (**O**) of protein expression following CNBP modulation. WB analyses (**P**) showed CNBP overexpression promoted the nuclear accumulation of β-catenin. In vivo (**Q-V**): CNBP overexpression promoted subcutaneous tumor growth (n=5 mice per group) (**Q-S**). Representative tumor image (**Q**), tumor weights (**R**), and tumor growth curves (**S**). CNBP knockdown inhibited subcutaneous tumor growth (n=5 mice per group) (**T-V**). Representative tumor image (**T**), tumor weights (**U**), and tumor growth curves (**V**). Data are expressed as mean ± SD. (Student′s t-test, *, p < 0.05, **, p < 0.01, and ***, p < 0.001).

**Supplementary Fig 3. A-B.** qRT-PCR validation of the top 11 candidate downstream genes in HGC-27 cells with tRF-Ser overexpression and knockdown. **C-F.** IHC (**C, D**) and qRT-PCR (**E, F**) assays of 30 pairs of GC and adjacent tissues showing high HSPA8 expression in GC. **G-I.** Validation of transfection efficiency for HSPA8 overexpression plasmid and siRNA by qRT-PCR (**G, H**) and WB (**I**) analyses. **J-O.** HSPA8 overexpression reversed the tumor-suppressive effects of tRF-Ser overexpression. Functional assays including CCK-8 (**J**), colony formation (**K, L**), Transwell invasion (**M, N**), and cell cycle analysis by flow cytometry (**O**) in GC cells. **P-R.** HSPA8 knockdown reversed the pro-tumorigenic effects of CNBP overexpression. Functional assays including colony formation (**P**) and cell cycle analysis by flow cytometry (**Q, R**) in GC cells. Data are expressed as mean ± SD. (Student′s t-test, **, p < 0.01, and ***, p < 0.001).

**Supplementary Fig 4. A-B.** Cell viability (CCK-8 assay) in MKN-45 (**A**) and HGC-27 (**B**) cells with tRF-Ser modulation after Erastin treatment. **C-D.** Changes in GSH (**C**) and MDA (**D**) levels with tRF-Ser overexpression or knockdown in MKN-45 and HGC-27 cells. **E-H.** tRF-Ser enhanced Erastin-induced (MKN-45: 10 μM, HGC-27: 4 μM) ROS accumulation in MKN-45 (**E, F**) and HGC-27 (**G, H**) cells. **I.** Representative TEM images of mitochondria in MKN-45 cells. tRF-Ser overexpression exacerbated Erastin-induced (10 μM) mitochondrial damage (e.g., shrinkage, loss of cristae), hallmarks of ferroptosis, while tRF-Ser knockdown attenuated this damage. **J.** WB analyses showed that tRF-Ser downregulated GPX4 protein expression. **K-Q.** Ferrostatin-1 reversed the pro-ferroptosis effects of tRF-Ser overexpression. Functional assays including WB (**K**), ROS levels (**L-O**), GSH levels (**P**), and MDA levels (**Q**) in GC cells. Data are expressed as mean ± SD. (Student′s t-test, *, p < 0.05, **, p < 0.01, and ***, p < 0.001).

**Supplementary Fig 5. A-C.** CNBP suppressed Erastin-induced (MKN-45: 10 μM, HGC-27: 4 μM)ROS accumulation. **D.** Representative TEM images of mitochondria in MKN-45 cells. CNBP knockdown exacerbated Erastin-induced (10 μM) mitochondrial damage (e.g., shrinkage, loss of cristae), while its overexpression attenuated it. **E-F.** CNBP overexpression increased GSH and decreased MDA levels, while knockdown had the opposite effects. **G-L.** Ferrostatin-1 reversed the pro-ferroptosis effects of CNBP knockdown. Functional assays including ROS levels (**G-J**), WB (**K**), GSH levels (**L**), and MDA levels (**M**) in GC cells. Data are expressed as mean ± SD. (Student′s t-test, *, p < 0.05, **, p < 0.01, and ***, p < 0.001).

**Supplementary Fig 6. A-F.** CNBP knockdown reversed the effects induced by tRF-Ser knockdown: ROS levels after Erastin induction (MKN-45: 10 μM, HGC-27: 4 μM) (**A-D**), GSH levels (**E**), and MDA levels (**F**). **G.** Representative TEM images of mitochondria in MKN-45 cells. CNBP knockdown exacerbated the mitochondrial damage induced by tRF-Ser knockdown under Erastin treatment (10 μM). **H-I.** HSPA8 overexpression reversed the changes in MDA (**H**) and GSH (**I**) levels induced by tRF-Ser overexpression in GC cells. **J-M.** HSPA8 overexpression reversed the increased ROS induced by tRF-Ser overexpression after Erastin treatment (MKN-45: 10 μM, HGC-27: 4 μM). Data are expressed as mean ± SD. (Student′s t-test, **, p < 0.01, and ***, p < 0.001).

**Supplementary Fig 7.** **A-B.** HSPA8 knockdown reversed the suppression of ROS by CNBP overexpression under Erastin treatment (10 μM) in MKN-45 cells. **C-E.** HSPA8 knockdown reversed the increase in GSH (**C, D**) and decrease in MDA (**E**) caused by CNBP overexpression. **F.** Representative TEM images of MKN-45 cells showed that HSPA8 knockdown exacerbated mitochondrial damage in CNBP overexpression cells after Erastin induction (10 μM). **G.** GPX4 ubiquitination assay showed that HSPA8 overexpression reduced the ubiquitination promoted by CNBP knockdown. **H.** GPX4 ubiquitination assay showed that CNBP knockdown increased the ubiquitination suppressed by tRF-Ser knockdown. **I-J.** tRF-Ser modulation altered GSH (**I**) and MDA (**J**) levels in HGC-27 cells treated with 5-FU (20 μM) + Erastin (4 μM). Data are expressed as mean ± SD. (Student′s t-test, **, p < 0.01, and ***, p < 0.001).

**Supplementary Figure 8.** Original WBs.

**Supplementary Table 1.** The primer sequences of the genes in this experiment.

| Genes | Primer sequences (5′ to 3′) |
| --- | --- |
| tRF-Ser | F: TGTCTAATGCGTCACGGTGG |
|  | R: TATGGTTGTTGACGACTGGTTGAC |
| U6 | F: CTCGCTTCGGCAGCACA |
|  | R: AACGCTTCACGAATTTGCGT |
| TXNIP | F: GGTCTTTAACGACCCTGAAAAGG |
|  | R: ACACGAGTAACTTCACACACCT |
| CHAC1 | F: GAACCCTGGTTACCTGGGC |
|  | R: CGCAGCAAGTATTCAAGGTTGT |
| CCDC18-AS1 | F: AAACTGTCGTCCTGGTGGG |
|  | R: CAGCGTAAGGGTGGAACAG |
| STC2 | F: ACAGGTTCGGCTGCATAAGC |
|  | R: GAGGTCCACGTAGGGTTCG |
| SPRED2 | F: CAAACTGGTGGTATTGGAATGCT |
|  | R: CAGCTTCATTATGGATGGTGGAA |
| DUSP1 | F: AGTACCCCACTCTACGATCAGG |
|  | R: GAAGCGTGATACGCACTGC |
| HSP90AB1 | F: CGAAGTTGGACAGTGGTAAAGAG |
|  | R: TGCCCAATCATGGAGATGTCT |
| EIF5A | F: GGACTTCGAGACAGGAGATGC |
|  | R: TCATTCCTTTTGATGTTGGGGAC |
| HSPA8 | F: ACCTACTCTTGTGTGGGTGTT |
|  | R: GACATAGCTTGGAGTGGTTCG |
| DNAJA1 | F: GACATACAGCTCGTTGAAGCA |
|  | R: GTGATGACGATGGTTCGGTTG |
| RBM3 | F: TGAGAGCCATGAACGGAGAGT |
|  | R: GTAGCGGTCATAACCACCCTG |
| CNBP | F: AAACTGGTCATGTAGCCATCAAC |
|  | R: AATTGTGCATTCCCGTGCAAG |
| β-actin | F: CATGTACGTTGCTATCCAGGC |
|  | R: CTCCTTAATGTCACGCACGAT |
| HSPA8-ChIP | F: TTCAAAGCGTCGGTATGGTCT |
|  | R: AGCCGTGCGTCCTTGATTTA |

**Supplementary Table 2.** Oligonucleotide sequences used in the cell transfection.

| Groups | Oligonucleotide sequences (5′ to 3′) |
| --- | --- |
| oe-tRF-Ser (mimics) | GUCACGGUGGCCGAGU |
| oe-NC (mimics-NC) | UCACAACCUCCUAGAAAGAGUAGA |
| in-tRF-Ser (inhibitor) | ACUCGGCCACCGUGAC |
| in-NC (inhibitor-NC) | UCUACUCUUUCUAGGAGGUUGUGA |
| si-CNBP-1 | AAUGAGAAGCCGUGGCAGATT |
| si-CNBP-2 | GCAAGGAGCCCAAGAGAGATT |
| si-CNBP-3 | UGGUGAAACUGGUCAUGUATT |
| si-CNBP-NC | UUCUCCGAACGUGUCACGUTT |
| si-HSPA8 | GCCCAAGGUCCAAGUAGAAUA |
| si-HSPA8-NC | UUCUCCGAACGUGUCACGU |

**Supplementary Table 3.** Sequences of the FISH probes.

| Groups | Sequences (5′ to 3′) |
| --- | --- |
| tRF-Ser | ACTCGGCCACCGTGAC |
| U6 | CACGAATTTGCGTGTCATCCTT |
| 18S | CTGCCTTCCTTGGATGTGGTAGCCGTTTC |

**Supplementary Table 4.** The dual-luciferase sequences of HSPA8 promoter both wild-type and mutant in pGL4.10

Sequence of HSPA8 promoter wild-type in pGL4.10.

| Sequence (5′ to 3′) |
| --- |
| AACCACAAGGCCCCACATATCCGCGGCCATGCACAACATTGCACAATTCAAAATTCTAGGCACCATTGTTTTGTGCACCAGCTCAGTCACGTTTTCAAAGCGTCGGTATGGTCTGTCGTTTAATTTTTGATGAAAGTCAAACCAGGAAAGATATCACCTCATGTTACTGAAAATACAAACGTTCAGAAAGTCTAAATCAAGGACGCACGGCTACTGGAACCTATGATTCCTAATGCCTGGTTCACCTAACCAAGAACACACTCGCCACCATTTAAACCTGTTTATTTTATAAACAACGGGGAAATGTAGGGCTTGGGCTAGTTGGGGAAGGACTGAGCCAAGAAGCCGAATCTGTTCTAGACGGAGGTGGGAAGGGGCCTAACGGTCAGGGCTCAGAGAAGCGGTTTCGCCTTCTTTTCCGCGGAGGGATCCAGACACGACCGCGTCCTGAGGCGGTTCTGAAAAGCCGGGCCCCTTAAACCTCTGCGTCGGGGGCTGCCTCCCGACAGTTGCCGTAGGGAAATGCAGGTGCTGGGAGGCCTGCCGAGCTAACCCGCCCCACCCCGCGGCGGCCTGGCGGCTCCCTCCAATCCCAATCCTGGGGGGCCGTGAGCGAGCAGCCCTAGTGGCACCCTGAAGCCGGAACCTTCGCCCCAGCCCCTCGGGGTCAGCCCTTGTCCAAAGCCACGCTATGAAATACAGATTCGCGAAACCCGGGGCACTTAGAGGCCCGTCTGGAACACGAAGGTGGGGCATGGAAAGGAGGATTCGGACGTGGGTGGGGGAGGGGAGCAACAACCTTCGCAGCCATTTTGTCCTCGCTCCACTTCCGTCTTCTCCTGCCCGGCTCCCGCGCCCAAACCCCTCCCTTCAGGCCCCGCGCGATTCCGCCCCCAGTTCTGTGCCGGCCAAGATCCCGGCTAGCGCCGCTATCATTGGTTAGTTCCAAGTTTGCCCGCCCCTCTTCCTCCTCCTTTTTCCGCCCCCTCCCTCCCGCGGAAGCTGGGGGCGCATGCGTAGAGGTGGACGCTCCCCTCCCCCGCCCGGGGTAACTGAGGACTCCCGCGCGCGGACTCGCTGCGCCCCACCCTCCCTTTCCCCGGGGCCGTCCGGAGAGCGGGGGCGAGCTTGAAAGTTCCAGAACGCTGCGGTGAGTGCGTTATCGTGAGGCGGAGCGCGGTGGGGTGGGTGCGGAAGGGGGCGAGGCCCGAGGAGTGGAGCCGGGCTTGTGATTGGGTCTTGTAAGGGCAGCCGGGCGTCTATTGGCCGGGGAAGCCGTAATGGCAGGCAGCAGGGGCGGGCCCCTTCTGGAAGGTTCTAAGATAGGGTATAAGAGGCAGGGTGGCGGGCGGAAACCGGT |

Sequence of HSPA8 promoter mutant in pGL4.10.

| Sequence (5′ to 3′) |
| --- |
| AACCACAAGGCCCCACATATCCGCGGCCATGCACAACATTGCACAATTCAAAATTCTAGGCACCATTGTTTTGTGCACCAGCTCAGTCACGTTTTCAAAGCGTCGGTATGGTCTGTCGTTTAATTTTTGATGAAAGTCAAACCAGGAAAGATATCACCTCATGTTAAGTCCCCGACAAACGTTCAGAAAGTCTAAATCAAGGACGCACGGCTACTGGAACCTATGATTCCTAATGCCTGGTTCACCTAACCAAGAACACACTCGCCACCATTTAAACCTGTTTATTTTATAAACAACGGGGAAATGTAGGGCTTGGGCTAGTTGGGGAAGGACTGAGCCAAGAAGCCGAATCTGTTCTAGACGGAGGTGGGAAGGGGCCTAACGGTCAGGGCTCAGAGAAGCGGTTTCGCCTTCTTTTCCGCGGAGGGATCCAGACACGACCGCGTCCTGAGGCGGTTCTGAAAAGCCGGGCCCCTTAAACCTCTGCGTCGGGGGCTGCCTCCCGACAGTTGCCGTAGGGAAATGCAGGTGCTGGGAGGCCTGCCGAGCTAACCCGCCCCACCCCGCGGCGGCCTGGCGGCTCCCTCCAATCCCAATCCTGGGGGGCCGTGAGCGAGCAGCCCTAGTGGCACCCTGAAGCCGGAACCTTCGCCCCAGCCCCTCGGGGTCAGCCCTTGTCCAAAGCCACGCTATGAAATACAGATTCGCGAAACCCGGGGCACTTAGAGGCCCGTCTGGAACACGAAGGTGGGGCATGGAAAGGAGGATTCGGACGTGGGTGGGGGAGGGGAGCAACAACCTTCGCAGCCATTTTGTCCTCGCTCCACTTCCGTCTTCTCCTGCCCGGCTCCCGCGCCCAAACCCCTCCCTTCAGGCCCCGCGCGATTCCGCCCCCAGTTCTGTGCCGGCCAAGATCCCGGCTAGCGCCGCTATCATTGGTTAGTTCCAAGTTTGCCCGCCCCTCTTCCTCCTCCTTTTTCCGCCCCCTCCCTCCCGCGGAAGCTGGGGGCGCATGCGTAGAGGTGGACGCTCCCCTCCCCCGCCCGGGGTAACTGAGGACTCCCGCGCGCGGACTCGCTGCGCCCCACCCTCCCTTTCCCCGGGGCCGTCCGGAGAGCGGGGGCGAGCTTGAAAGTTCCAGAACGCTGCGGTGAGTGCGTTATCGTGAGGCGGAGCGCGGTGGGGTGGGTGCGGAAGGGGGCGAGGCCCGAGGAGTGGAGCCGGGCTTGTGATTGGGTCTTGTAAGGGCAGCCGGGCGTCTATTGGCCGGGGAAGCCGTAATGGCAGGCAGCAGGGGCGGGCCCCTTCTGGAAGGTTCTAAGATAGGGTATAAGAGGCAGGGTGGCGGGCGGAAACCGGT |

**Supplementary Table 5.** Sequences of the biotin-labeled RNA pull-down probes.

| Groups | Sequences (5′ to 3′) |
| --- | --- |
| tRF-Ser | GUCACGGUGGCCGAGU |
| antisense | ACUCGGCCACCGUGAC |

**Supplementary Table 6.** The specific tRF-Ser binding proteins identified by mass spectrometry are shown.

| Number | Accession | Genes | Coverage (%) | Score | PSMs | Unique Peptides | Molecular Weight (kDa) | calc. pI |
| --- | --- | --- | --- | --- | --- | --- | --- | --- |
| 1 | U3PXP0 | HBA2 | 29.41 | 52.06 | 1 | 1 | 5.7 | 7.52 |
| 2 | P35321 | SPRR1A | 17.98 | 29.99 | 2 | 1 | 9.9 | 8.48 |
| 3 | P59665 | DEFA1 | 9.57 | 35.34 | 2 | 1 | 10.2 | 6.99 |
| 4 | Q9BYR8 | KRTAP3-1 | 24.49 | 74.46 | 3 | 2 | 10.5 | 6.4 |
| 5 | Q5BQ95 | KLK13 | 8.16 | 25.3 | 1 | 1 | 10.7 | 9.69 |
| 6 | P01040 | CSTA | 14.29 | 70.37 | 2 | 1 | 11 | 5.5 |
| 7 | D6R904 | TPM3 | 13.68 | 26.43 | 2 | 2 | 11 | 4.79 |
| 8 | P0CG05 | IGLC2 | 9.43 | 77.38 | 2 | 1 | 11.3 | 7.24 |
| 9 | P62805 | HIST1H4A | 29.13 | 87.74 | 8 | 3 | 11.4 | 11.36 |
| 10 | P31151 | S100A7 | 10.89 | 37.01 | 2 | 1 | 11.5 | 6.77 |
| 11 | P05387 | RPLP2 | 10.43 | 28.95 | 2 | 1 | 11.7 | 4.54 |
| 12 | P10599 | TXN | 10.48 | 29.52 | 2 | 1 | 11.7 | 4.92 |
| 13 | H0YB22 | RPS14 | 9.17 | 33.85 | 1 | 1 | 12.9 | 9.85 |
| 14 | H7BZJ3 | PDIA3 | 20.33 | 38.9 | 2 | 2 | 13.5 | 7.3 |
| 15 | P0C0S5 | H2AFZ | 7.03 | 26.97 | 2 | 1 | 13.5 | 10.58 |
| 16 | P23527 | HIST1H2BO | 26.98 | 250.04 | 17 | 4 | 13.9 | 10.32 |
| 17 | P68431 | HIST1H3A | 8.09 | 23.55 | 2 | 1 | 15.4 | 11.12 |
| 18 | P62249 | RPS16 | 6.85 | 25.15 | 2 | 1 | 16.4 | 10.21 |
| 19 | Q15828 | CST6 | 7.38 | 24.62 | 2 | 1 | 16.5 | 8.09 |
| 20 | J3KRG2 | GSDMA | 5.06 | 20.39 | 1 | 1 | 17.8 | 6.02 |
| 21 | P62633 | CNBP | 4.52 | 27.34 | 2 | 1 | 19.4 | 7.71 |
| 22 | A0JLU4 | YBX1 | 13.26 | 21.37 | 1 | 1 | 21.4 | 10.33 |
| 23 | P28072 | PSMB6 | 4.6 | 49.18 | 2 | 1 | 25.3 | 4.92 |
| 24 | Q86V81 | ALYREF | 4.28 | 204.22 | 14 | 1 | 26.9 | 11.15 |
| 25 | Q9UKD2 | MRTO4 | 4.18 | 26.23 | 1 | 1 | 27.5 | 8.29 |
| 26 | P31947 | SFN | 14.11 | 106.5 | 7 | 3 | 27.8 | 4.74 |
| 27 | P62258 | YWHAE | 8.63 | 81.52 | 7 | 2 | 29.2 | 4.74 |
| 28 | P29692 | EEF1D | 4.27 | 67.97 | 6 | 1 | 31.1 | 5.01 |
| 29 | Q13243 | SRSF5 | 3.31 | 34.53 | 2 | 1 | 31.2 | 11.59 |
| 30 | P47756 | CAPZB | 3.61 | 23.28 | 2 | 1 | 31.3 | 5.59 |
| 31 | P45880 | VDAC2 | 7.48 | 49.46 | 4 | 2 | 31.5 | 7.56 |
| 32 | P47755 | CAPZA2 | 3.5 | 45.03 | 2 | 1 | 32.9 | 5.85 |
| 33 | P36542 | ATP5C1 | 7.05 | 47.71 | 3 | 2 | 33 | 9.22 |
| 34 | P22087 | FBL | 6.85 | 51.82 | 6 | 3 | 33.8 | 10.18 |
| 35 | P05089 | ARG1 | 7.14 | 65.93 | 6 | 3 | 34.7 | 7.21 |
| 36 | Q96AG4 | LRRC59 | 3.26 | 27.41 | 2 | 1 | 34.9 | 9.57 |
| 37 | Q9H9B4 | SFXN1 | 12.42 | 72.06 | 10 | 4 | 35.6 | 9.07 |
| 38 | Q13347 | EIF3I | 7.38 | 63.84 | 4 | 2 | 36.5 | 5.64 |
| 39 | P00338 | LDHA | 3.61 | 63.67 | 2 | 1 | 36.7 | 8.27 |
| 40 | P31942 | HNRNPH3 | 5.2 | 30.89 | 2 | 2 | 36.9 | 6.87 |
| 41 | P48059 | LIMS1 | 3.69 | 66.4 | 2 | 1 | 37.2 | 8.05 |
| 42 | Q15365 | PCBP1 | 5.62 | 41.63 | 4 | 2 | 37.5 | 7.09 |
| 43 | Q9H0U3 | MAGT1 | 2.69 | 38.33 | 2 | 1 | 38 | 9.63 |
| 44 | P04075 | ALDOA | 11.81 | 88.92 | 7 | 4 | 39.4 | 8.09 |
| 45 | P01860 | IGHG3 | 22.55 | 258.64 | 32 | 1 | 41.3 | 7.9 |
| 46 | P29508 | SERPINB3 | 6.67 | 79.43 | 7 | 3 | 44.5 | 6.81 |
| 47 | O75874 | IDH1 | 3.14 | 36.7 | 2 | 1 | 46.6 | 7.01 |
| 48 | Q8IW75 | SERPINA12 | 1.93 | 20.49 | 2 | 1 | 47.1 | 9.29 |
| 49 | Q15323 | KRT31 | 12.02 | 137.23 | 20 | 1 | 47.2 | 4.88 |
| 50 | O76011 | KRT34 | 9.63 | 81.88 | 15 | 2 | 49.4 | 5.06 |
| 51 | Q6KB66 | KRT80 | 4.65 | 53.43 | 4 | 1 | 50.5 | 5.67 |
| 52 | O43790 | KRT86 | 24.49 | 351.22 | 32 | 6 | 53.5 | 5.66 |
| 53 | Q15233 | NONO | 6.79 | 66.79 | 5 | 2 | 54.2 | 8.95 |
| 54 | P04217 | A1BG | 1.41 | 37.92 | 3 | 1 | 54.2 | 5.86 |
| 55 | P78386 | KRT85 | 21.7 | 345.95 | 32 | 6 | 55.8 | 6.55 |
| 56 | Q8N1N4 | KRT78 | 5.38 | 57.02 | 9 | 1 | 56.8 | 6.02 |
| 57 | P19013 | KRT4 | 5.24 | 177.67 | 18 | 1 | 57.2 | 6.61 |
| 58 | P25705 | ATP5A1 | 3.07 | 24.2 | 2 | 2 | 59.7 | 9.13 |
| 59 | Q6UWP8 | SBSN | 1.86 | 28.78 | 2 | 1 | 60.5 | 7.01 |
| 60 | Q9NSB2 | KRT84 | 7.67 | 125.69 | 26 | 2 | 64.8 | 7.56 |
| 61 | Q58F26 | DHX9 | 2.01 | 45.11 | 1 | 1 | 67.1 | 5.72 |
| 62 | Q8WVV4 | POF1B | 1.7 | 20.92 | 2 | 1 | 68 | 6.32 |
| 63 | P23246 | SFPQ | 1.41 | 33.84 | 3 | 1 | 76.1 | 9.44 |
| 64 | Q08188 | TGM3 | 3.17 | 51.41 | 4 | 2 | 76.6 | 5.86 |
| 65 | P0CG48 | UBC | 11.82 | 61.2 | 4 | 1 | 77 | 7.66 |
| 66 | P02788 | LTF | 1.27 | 117.55 | 10 | 1 | 78.1 | 8.12 |
| 67 | P14923 | JUP | 3.89 | 78.77 | 6 | 2 | 81.7 | 6.14 |
| 68 | Q13835 | PKP1 | 1.47 | 49.66 | 2 | 1 | 82.8 | 9.13 |
| 69 | P22735 | TGM1 | 1.1 | 21.74 | 2 | 1 | 89.7 | 6.04 |
| 70 | Q08554 | DSC1 | 6.82 | 47.34 | 8 | 5 | 99.9 | 5.43 |
| 71 | Q9H0D6 | XRN2 | 1.26 | 51.52 | 2 | 1 | 108.5 | 7.47 |
| 72 | Q86YZ3 | HRNR | 6.21 | 176.51 | 16 | 6 | 282.2 | 10.04 |
