# Supplementary figures and images for: A new candidate tumor suppressor tRF-Ser inhibits gastric cancer progression by regulating the CNBP/HSPA8 axis
Source: Cell Death Dis. 2026 Mar 25;17(1):379. doi: 10.1038/s41419-026-08608-1 (PMC13039117; doi:10.1038/s41419-026-08608-1)

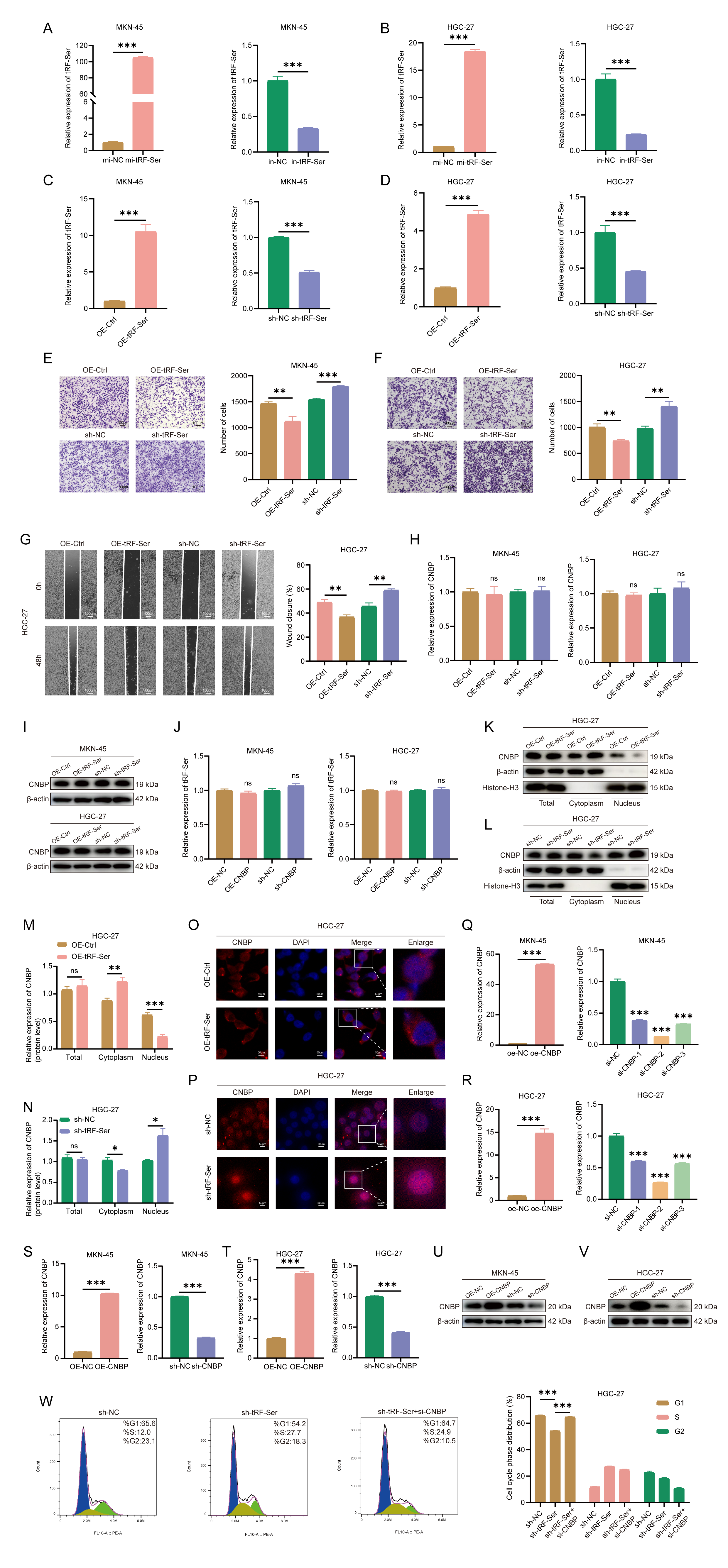

Supplement: Supplementary file 2 — Supplementary Figure 1 [file 41419_2026_8608_MOESM2_ESM.png]

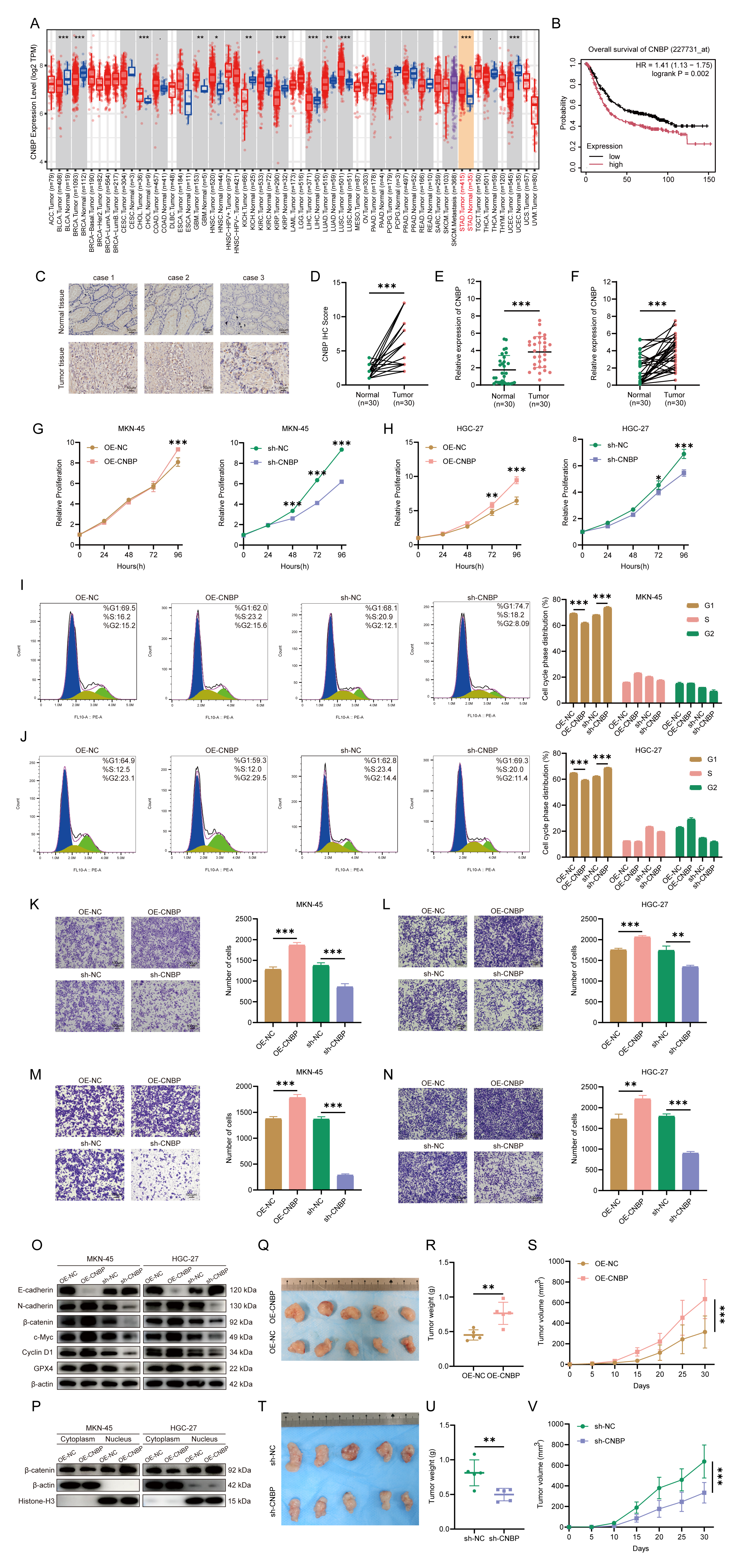

Supplement: Supplementary file 3 — Supplementary Figure 2 [file 41419_2026_8608_MOESM3_ESM.png]

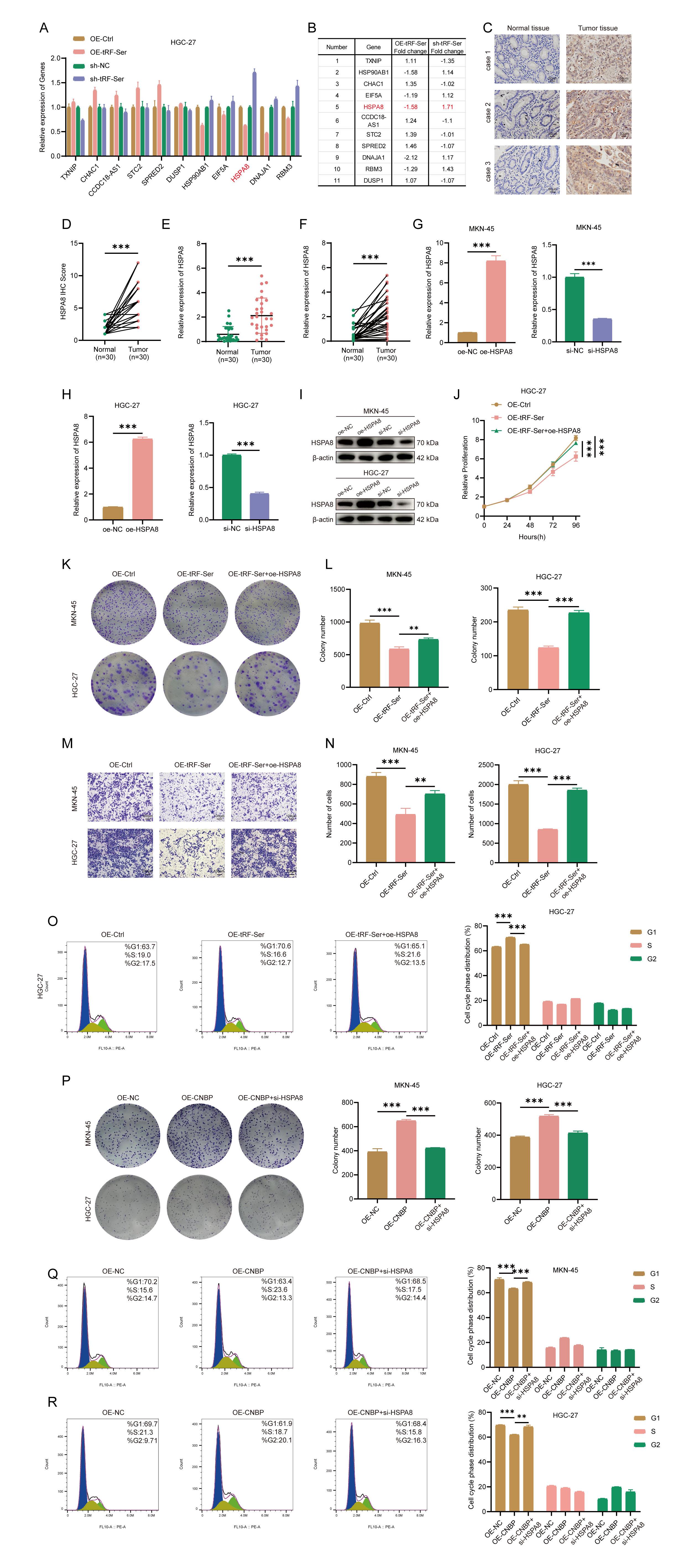

Supplement: Supplementary file 4 — Supplementary Figure 3 [file 41419_2026_8608_MOESM4_ESM.png]

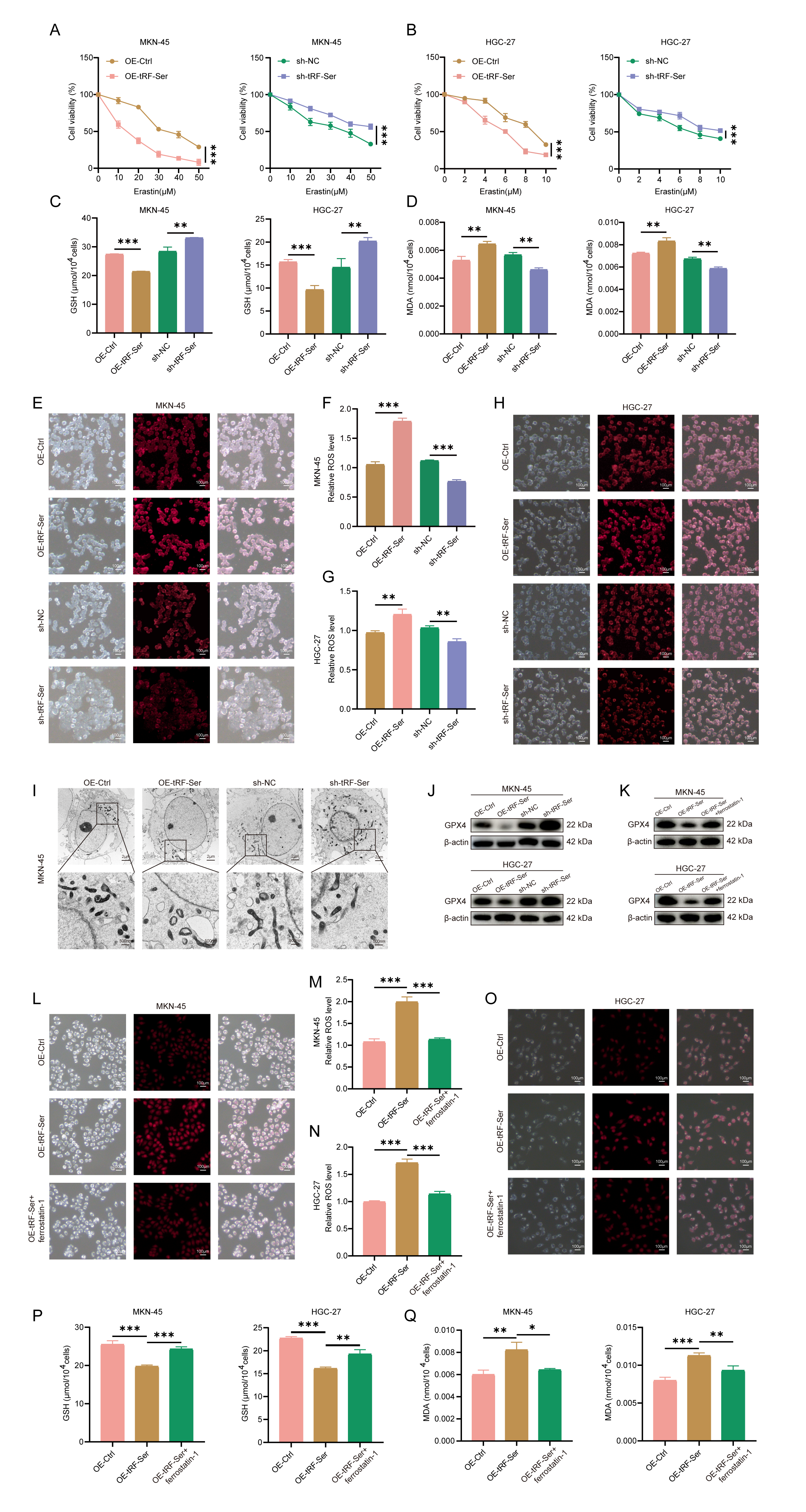

Supplement: Supplementary file 5 — Supplementary Figure 4 [file 41419_2026_8608_MOESM5_ESM.png]

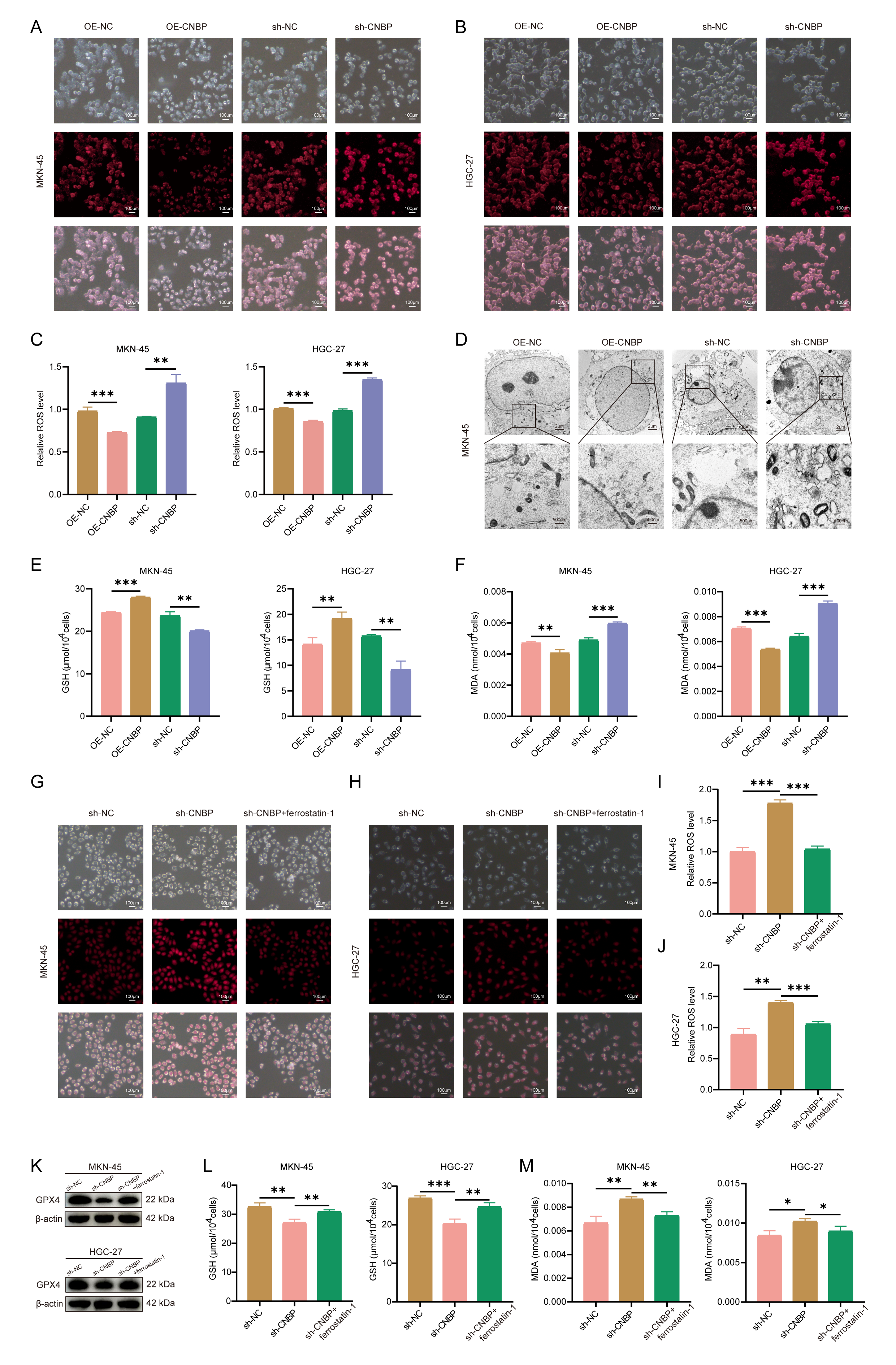

Supplement: Supplementary file 6 — Supplementary Figure 5 [file 41419_2026_8608_MOESM6_ESM.png]

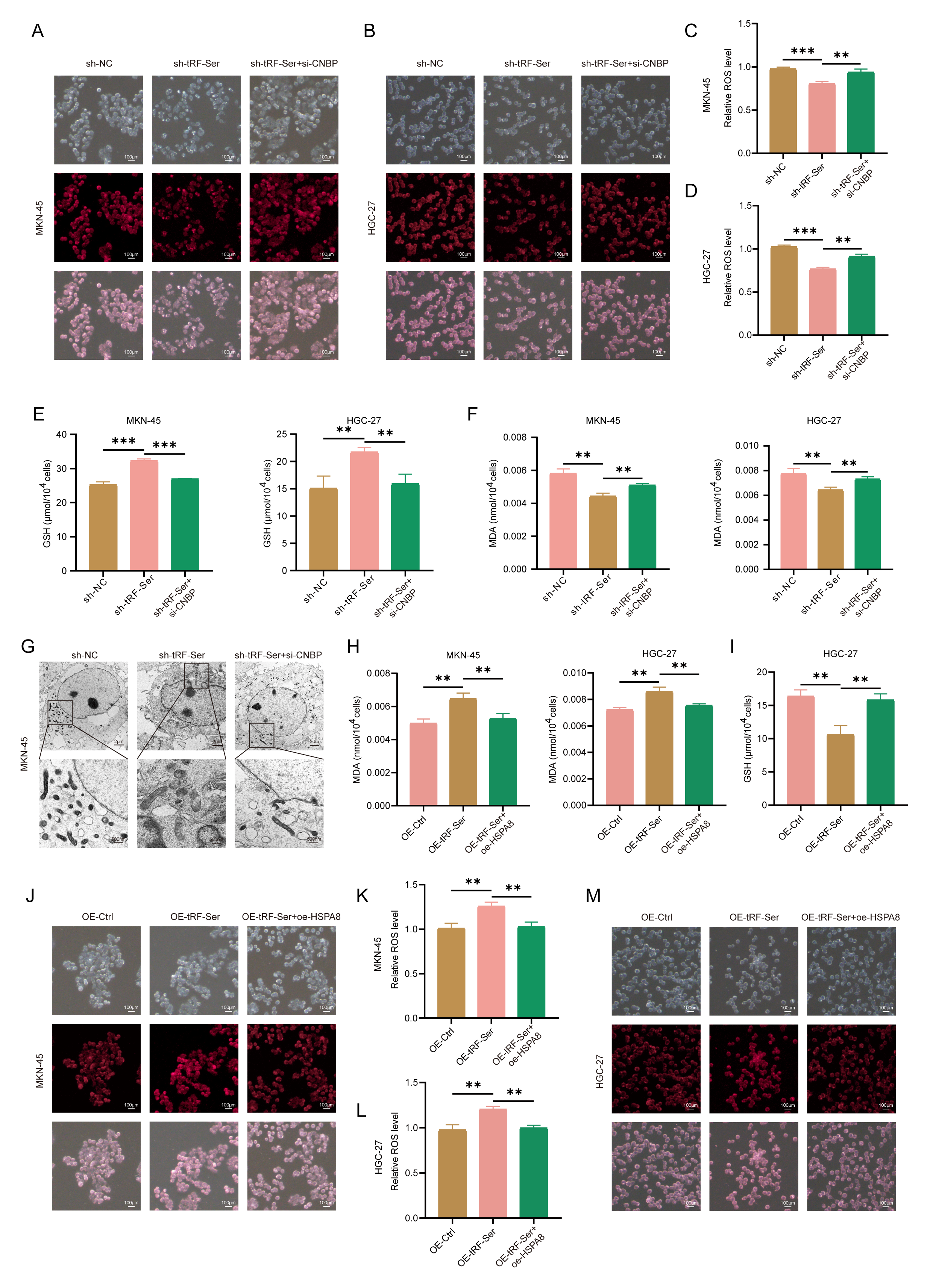

Supplement: Supplementary file 7 — Supplementary Figure 6 [file 41419_2026_8608_MOESM7_ESM.png]

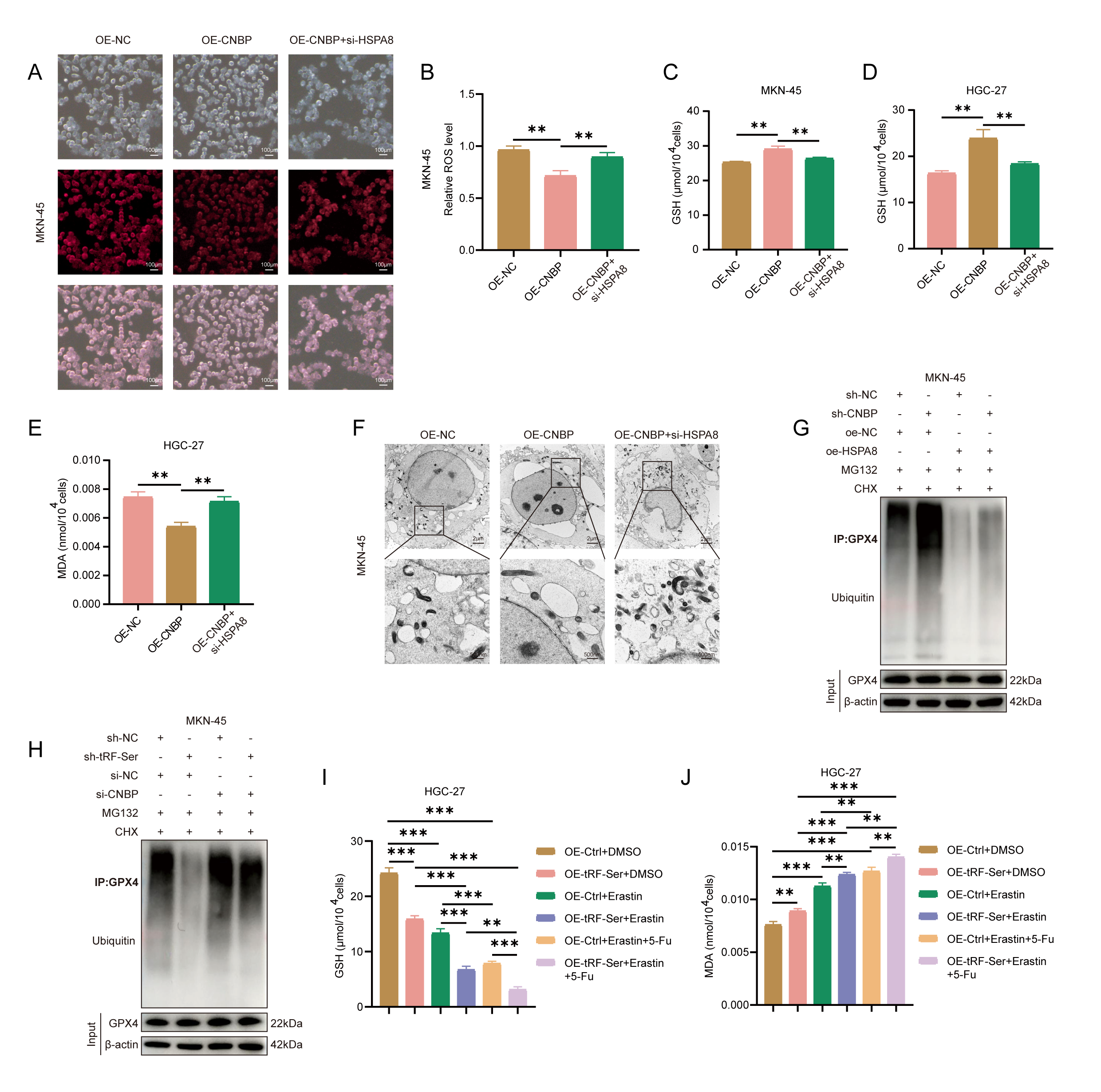

Supplement: Supplementary file 8 — Supplementary Figure 7 [file 41419_2026_8608_MOESM8_ESM.png]

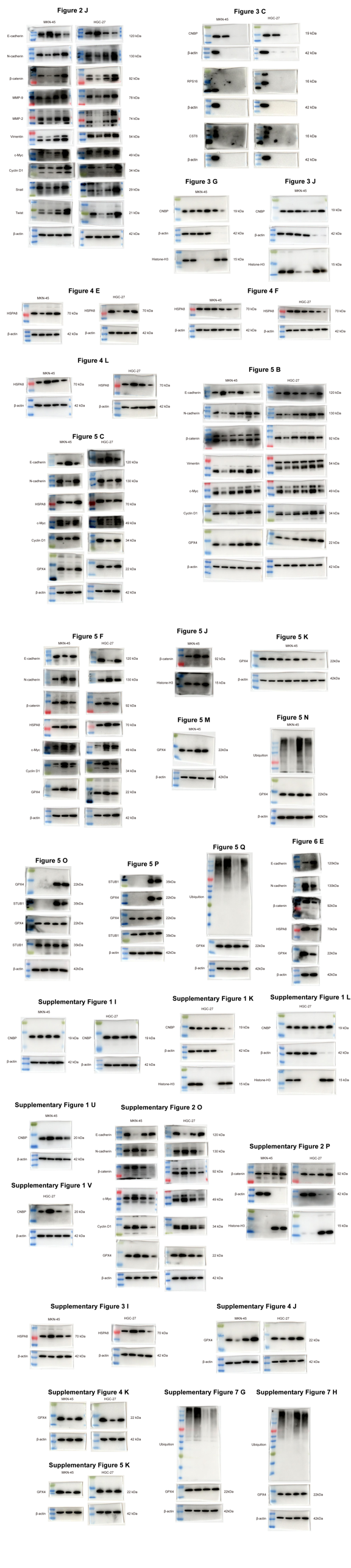

Supplement: Supplementary file 9 — Supplementary Figure 8 [file 41419_2026_8608_MOESM9_ESM.png]
